# Regrettable Substitutes and the Brain: What Animal Models and Human Studies Tell Us about the Neurodevelopmental Effects of Bisphenol, Per- and Polyfluoroalkyl Substances, and Phthalate Replacements

**DOI:** 10.3390/ijms25136887

**Published:** 2024-06-23

**Authors:** Elena Morales-Grahl, Emily N. Hilz, Andrea C. Gore

**Affiliations:** Division of Pharmacology and Toxicology, The University of Texas at Austin, Austin, TX 78712, USA; moralesgrahle@utexas.edu (E.M.-G.); ehilz@utexas.edu (E.N.H.)

**Keywords:** endocrine-disrupting chemical (EDC), neurodevelopment, PFAS, phthalate, bisphenol, BPA, bio-based plastic, zebrafish, rodent, human

## Abstract

In recent decades, emerging evidence has identified endocrine and neurologic health concerns related to exposure to endocrine-disrupting chemicals (EDCs), including bisphenol A (BPA), certain per- and polyfluoroalkyl compounds (PFASs), and phthalates. This has resulted in consumer pressure to remove these chemicals from the market, especially in food-contact materials and personal care products, driving their replacement with structurally or functionally similar substitutes. However, these “new-generation” chemicals may be just as or more harmful than their predecessors and some have not received adequate testing. This review discusses the research on early-life exposures to new-generation bisphenols, PFASs, and phthalates and their links to neurodevelopmental and behavioral alterations in zebrafish, rodents, and humans. As a whole, the evidence suggests that BPA alternatives, especially BPAF, and newer PFASs, such as GenX, can have significant effects on neurodevelopment. The need for further research, especially regarding phthalate replacements and bio-based alternatives, is briefly discussed.

## 1. Introduction

Bisphenols, phthalate esters, and per- and polyfluoroalkyl substances (PFASs) have been used widely for industrial and manufacturing purposes for nearly a century [1,2]. The term “endocrine-disrupting chemical” (EDC) was first introduced in the 1970s, when increasing evidence identified adverse reproductive and hormonal effects of these and other similarly-acting chemicals in humans and wildlife [3]. Some of these EDCs, known as “legacy” EDCs, persist in the environment and continue to be found in tissues of humans and wildlife despite being decades removed from commercial production [4,5,6,7]. Since then, public interest groups, regulatory bodies, and industry have pushed to ban or phase out the manufacture and use of certain chemicals. While this represents progress in terms of advancing toxicological safety standards to better address the unique properties of endocrine disruptors (which are not always identified via standard toxicology testing) and reducing human exposure to legacy EDCs, a concerning trend has emerged wherein these chemicals are replaced in consumer products or in manufacturing with sister compounds that are structurally or functionally similar [8,9,10]. The result is a phenomenon termed “regrettable substitution”, by which a compound is replaced with a substitute chemical that may later prove to have a similar or even stronger endocrine-disrupting capability—often after it has been released into the market. 

The developing brain is a target of EDCs, with far-reaching consequences on an individual’s mental and neurological health and wellness. The brain has high hormone sensitivity, especially as it undergoes rapid growth during critical periods of development including the prenatal period, infancy, and adolescence. During this time, steroid hormones such as androgens and estrogens organize the brain in a sexually dimorphic manner and later activate certain sex-specific brain processes [11,12,13]. EDCs can interfere with this by altering the normal function of these and other hormones, acting as hormone mimics, blockers, or altering synthesis and transport [14,15,16,17,18]. The disruption of hormone signaling during critical periods can have lasting effects on brain structure and function, potentially affecting later cognitive–behavioral function and increasing the risk of neurodevelopmental disorders such as autism spectrum disorder, attention deficit/hyperactivity disorder (ADHD), and/or learning disabilities in humans and animal models [19,20,21,22,23]. 

Legacy EDCs such as bisphenol A (BPA) and certain phthalate esters [i.e., butyl benzyl phthalate (BBP), (2-ethylhexyl) phthalate (DEHP), and dibutyl phthalate (DBP), among others] alter the development of the brain and lead to disrupted cognitive–behavioral function in adulthood in zebrafish, rodents, and humans [24,25]. The same is true for legacy PFASs [26,27]. As the brain is highly sensitive to hormones and serves as a central regulatory hub for many of the body’s homeostatic functions, disrupted development has implications beyond cognitive and affective behavior, affecting a range of functions such as metabolic, sexual, and immunological health [15,18,28]. This and other scientific evidence have inspired the phasing out of such chemicals; however, new-generation bisphenols, PFASs, and phthalate esters have emerged to take their place. Because the regulatory framework that allowed the mass use of legacy EDCs also grandfathered in new-generation substitutes, there is a real concern in the scientific community that “like has replaced like” and that new-generation substitutes do not represent a lessened toxicological burden. Rather, these substitutes lack the scientific, public, and regulatory scrutiny of their predecessors and represent a new cadre of EDCs whose toxicological profile has yet to be determined [8,29,30,31]. 

This review collects available information on new-generation bisphenols, PFASs, and phthalate esters in the context of the developing brain. Because of the rise in bio-based alternatives such as bioplastics, we also discuss the limited evidence for their possible endocrine-disrupting activity. Here, we discuss the literature on neurodevelopmental disruption from several model organisms—zebrafish, rodents, and humans—focusing on cognitive and other neurobiological consequences. All articles discussed and cited were found on PubMed prior to March 2024 using combinations of the following search terms: bisphenol/per- and polyfluoroalkyl substance/phthalate/bio-plastic AND neuro*/behavior/replacement AND rodent/mice/rat/zebrafish/human. Only studies using in vivo models (zebrafish, mice, rats, and humans) of single-chemical exposure are discussed. Studies that only examined legacy EDCs (e.g., BPA) and no replacement or substitute EDC (as discussed below) were excluded. We also excluded studies whose EDC exposures started after 30 dpf in zebrafish, P21 in rodents, and 13 years of age in humans, as we were interested in earlier periods of development. We verified that all included studies appeared to be appropriately powered and used proper controls. 

## 2. Legacy EDCs and New-Generation Substitutes

### 2.1. Bisphenols

**Figure 1:**
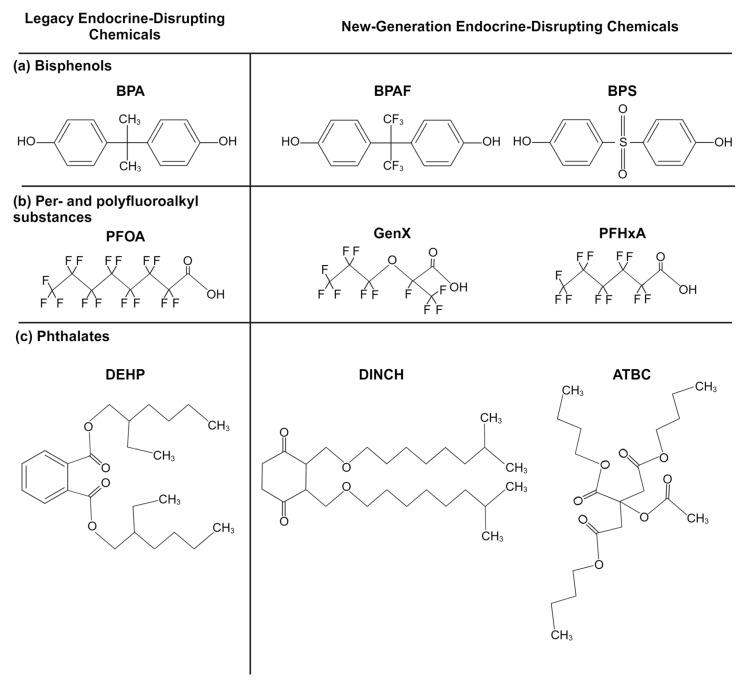
Example chemical structures of (**a**) bisphenol, (**b**) per- and polyfluoroalkyl substance, and (**c**) phthalate legacy and new-generation endocrine disruptors (created with BioRender.com (accessed on 20 June 2024)).

Bisphenols are a group of chemicals made up of two phenol rings joined by an atom, often carbon (Figure 1a). They are used to make epoxy resins, acting as protective coatings in metal products, such as pipes and food cans. Bisphenols are also polymerized to make polycarbonate, a hard plastic found in everyday products, from electronics to food containers [32]. The most well-known member of this group, bisphenol A (BPA), underwent selective bans around the world in the 2000s, especially in baby bottles, after research studies linked the chemical to cancers, diabetes, obesity, and reproductive and neurodevelopmental problems and attracting public outcry [32,33,34,35,36,37]. The “BPA-free” label has since become near-ubiquitous. This label is deceptive, however, as it does not reveal that BPA has been replaced with newer analogs whose safety may not be thoroughly understood (see Table 1 for bisphenol substitute examples). Evidence suggests that some of these BPA analogues may be as or more harmful than BPA as endocrine-disrupting chemicals, acting as carcinogens, obesogens, and more [38,39,40,41]. 

The mechanisms of action of bisphenols are varied, with evidence showing they can act on estrogen receptors [38,42], androgen receptors [38,43], thyroid hormone receptors [44], glucocorticoid receptors [45], and peroxisome proliferator-activated receptor γ (PPARγ) [46]. Any or all of these are potentially relevant to neurodevelopment and behavior as these receptors are expressed in the brain [47]. Recently, certain G-protein-coupled receptors were identified as being activated by plastics including BPA and diethyl phthalate [48]. Although the half-lives of bisphenols are short, their persistence in the environment yields detectable levels in most humans. The detection of BPA in humans is decreasing while the detection of BPA alternatives, such as BPF and BPS, is increasing [39]. 

### 2.2. Per- and Polyfluoroalkyl Substances

Per- and polyfluoroalkyl substances (PFASs) are a group of >9000 chemicals made up of a carbon–fluorine backbone and a polar functional group (Figure 1b). One of the most famous PFASs, polytetrafluoroethylene, known under its trademarked name, Teflon, is used as a nonstick coating for cookware. PFASs’ nonstick, waterproof, and stain-repellent properties drove their wide utilization in food packaging, clothing (e.g., Gore-Tex), furniture (e.g., Scotchgard, Stainmaster treatments), and even fire-suppressing foams [49,50]. Their thermal/chemical stability and amphiphilicity unfortunately also increase their persistence both in the environment and in the body [51,52]. PFAS exposure has been linked with cancer, altered immune function, reproductive deficits, and other adverse health outcomes [53,54,55,56]. PFAS interferes with the thyroid hormones and brain-derived neurotrophic factor and leads to an increase in oxidative stress and cell apoptosis, all of which are involved in neurodevelopment [31]. Because of the wide body of research demonstrating the dangers of PFASs, PFOS, PFOA, and PFHxS were globally banned under the Stockholm Convention in 2009, 2019, and 2022, respectively [57]. Although now members of the legacy chemical family, their persistence makes them a lasting part of the world’s ecosystem.

Fluorinated chain length is one of the key factors considered regarding PFAS safety, as longer chain lengths are typically associated with higher toxicity and biomagnification [52,58,59]. Because of this, there has been a rise in short-chain alternatives (≤4 continuous fluorinated carbons), including GenX (hexafluoropropylene-oxide-dimer-acid) and ADONA (dodecafluoro-3H-4,8-dioxanonanoate) [60]. Unfortunately, research indicates that these alternatives may not be any safer [55,56,61]. In addition, long-chain PFAS concentrations continue to increase in living organisms, a trend that will likely continue thanks to their ubiquitousness, due to their accumulation in the food chain and their release from melting ice caps [61,62]. In this review, we will focus on short- and long-chain PFASs that are not the legacy chemicals PFOS, PFOA, and PFHxS (see Table 1 for examples) and that are not as well evaluated and may prove to be “regrettable substitutions”.

### 2.3. Phthalates

Phthalates (also referred to as ortho-phthalate diesters and phthalic acid esters) are esters of phthalic acid, 1,2-benzenedicarboxylic acid (Figure 1c). They are primarily used as plasticizers for cosmetic products, medical devices, toys, and food packaging. Phthalate exposure has been linked to effects on the neuroendocrine and reproductive systems and neurodevelopment, as well as to increases in cancer, asthma, diabetes, and other health concerns [37,63]. Their effects on neurodevelopment are of special concern given the extensive phthalate exposure that premature and intensive care newborns are exposed to through medical tubing [64]. In 2017, the United States banned eight phthalates from use in children’s products, including toys. These included diisononyl phthalate (DINP), di(2-ethylhexyl) phthalate (DEHP), di-n-butyl phthalate (DBP), and diisobutyl phthalate (DIBP) [65]. Advocacy groups petitioned the FDA to ban the use of phthalates in food packaging, but in 2022, this petition was denied [66]. Efforts to create safer options resulted in the development of structurally similar groups, including isophthalates, terephthalates, and trimellitates, advertised as “non-phthalate alternatives”. Structurally distinct alternatives now exist as well. The most common alternatives include Bis(2-ethylhexyl) terephthalate (DEHT), diethylhexyl adipate (DEHA), and 1,2-cyclohexane dicarboxylic acid diisononyl ester (DINCH) [67]. In this paper, we focus on alternatives to ortho-phthalates and their impacts on neurodevelopment (Table 1). However, there is little research assessing the endocrine- and neurodevelopmental-disrupting potential of phthalate replacements. Research studies do suggest that DINCH, for example, is not estrogenic or androgenic, although it can disrupt steroidogenesis [68,69]. DINCH induces oxidative stress in some cell lines, causing potential disruptions in neurodevelopment [70,71].

## 3. Models of Neurodevelopment

### 3.1. Zebrafish

Zebrafish are useful models for studying neurodevelopment and neurotoxicity. Thanks to their small size, high breeding rate, and short life cycle, they are a high-throughput screening tool that enables researchers to predict the potential neurotoxicity of chemicals in mammals [72].

The neural signaling systems of zebrafish are homologous to humans, including the GABAergic, dopaminergic, and glutamatergic systems. The presence of a blood–brain barrier allows for the assessment of chemical entry into the brain. Through immunostaining techniques that allow for the direct assessment of neural growth and death, in addition to the widespread analysis of gene expression patterns, zebrafish are also useful for understanding the mechanisms of neurotoxicity.

Multiple zebrafish behavioral endpoints can be used to understand the neurobehavioral effects of endocrine-disrupting chemicals. Behaviors measured include embryonic spontaneous movements and larvae locomotion, where changes can indicate developmental motor–neural toxicity. Selderslaghs et al. looked at a wide set of known neurotoxic and non-neurotoxic chemicals and found that 9/10 of the chemicals tested demonstrated developmental neurotoxicity concordance between mammal studies and zebrafish studies assessing embryonic and larvae movement. Newer studies are also adapting classical rodent assays to zebrafish models, including the open-field assay, T-maze, and social tests to assess more complex behaviors such as anxiety, learning, reward preference, and sociability [73,74,75].

**Figure 2:**
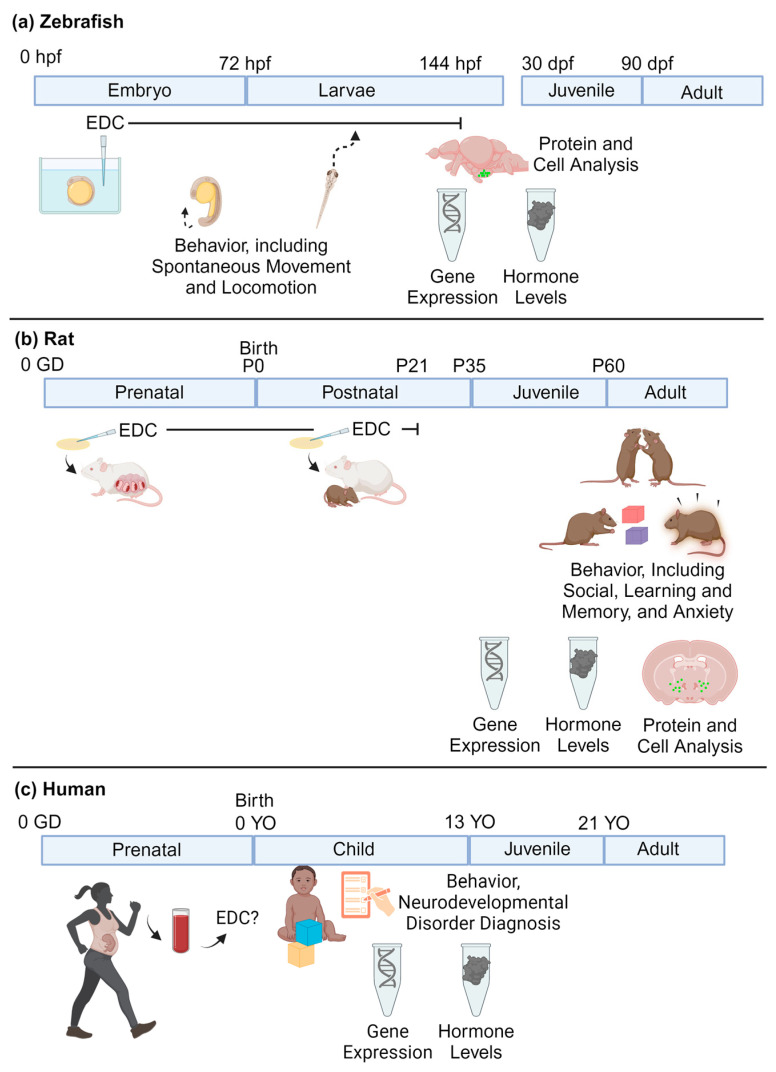
(**a**) Zebrafish, (**b**) rat, and (**c**) human models of neurodevelopmental exposure to EDCs. In a and b, EDC exposures are controlled and typically given through prenatal and/or postnatal exposure. In rats, this typically ends at or before weaning at P21. Note that the procedures in mice and rats are similar. In humans, exposures will vary across individual subjects. hpf = hours post-fertilization; dpf = days post-fertilization; GD = gestational days; P = postnatal days; YO = years old (created with BioRender.com (accessed on 5 April 2024)).

For neurodevelopmental research, zebrafish embryos are placed in well plates filled with the chemical of choice in DMSO, or another solvent. The liquid exposure media will commonly be changed throughout the duration of the exposure, which can range from a couple of hours to days. Zebrafish often undergo behavioral and molecular testing at numerous developmental stages (Figure 2a). 

### 3.2. Rodents

As mammals, rodents share more similarities than zebrafish to humans when it comes to brain structure, physiology, and behavior [76,77]. Unlike humans, rats can undergo experimentation, allowing for the causality of the effects of EDC exposures to be determined. Homologous endocrine systems in rodents and humans also allow for the analysis of the sexually dimorphic effects of toxicants on neurodevelopment.

A wide array of tests in rodents aim to assess human-relevant behaviors, including assays for anxiety, learning and memory, and social and sexual behaviors. Many of these tests show concordance with human toxicological effects [78]. Well-established antibodies and probes allow scientists to look at specific brain regions, cell types, and markers. Advances in genetic and transcriptomic analysis in rodents also aid in understanding the effects and potential mechanisms of toxicants on the central nervous.

Rodent chemical exposures are most typically administered via oral consumption (e.g., feeding a wafer or other edible treated with the chemical, or gavage, the latter which is more stressful), drinking water treated with the chemical, or via injection. For perinatal exposures, this will often consist of administering the chemical to the dam during gestation and lactation rather than subjecting developing rodents to injections or gavage. The offspring then undergo behavioral and molecular testing at a variety of selected ages from early postnatal life through adulthood (Figure 2b).

### 3.3. Humans

Studies assessing the effect of toxicants on humans are nearly always correlational, with the exception of known chemical spills or certain industrial settings. Although researchers do their best to include confounding variables, such as maternal education, smoking, and socio-economic status, the nature of this research means causal effects of toxicants cannot be elucidated in the general population. 

For human research studies, blood or urine samples may be collected from the mother during pregnancy, from the umbilical cord at birth, and/or from the child. Amniotic fluid can also be used as a proxy of exposure. For neurodevelopmental assessments, the children undergo a test of choice (e.g., IQ, autism spectrum disorders, ADHD assessments, social behaviors), and a statistical analysis is conducted to explore whether higher chemical concentrations are associated with scores on the given tests (Figure 2c).

## 4. Bisphenol Alternatives and Neurodevelopment

### 4.1. Bisphenols and Zebrafish

Embryonic and larval BPA analogue exposure has neurotoxic effects in zebrafish (Table 2). In general, embryonic and larval exposure to bisphenols decreases embryo spontaneous movement and larvae locomotion [26,74,79,80,81,82,83,84,85,86,87], with the exception of BPS, which produced mixed results. According to multiple comparison studies, BPAF leads to the most severe alterations in locomotion, followed by BPF, which is similar to BPA, and then BPS [80,81]. Open-field test results did not show consistent results [74,87,88]. Instances of social behavior decreased following perinatal exposure to BPAF, BPAP, BPB, BPC, BPF, BPS, and HPP [73,89]. Studies also suggest deficits in learning and memory (BPS) [88] and response to stimuli (BPF, BPS) [87,90]. 

It is clear from the zebrafish studies that exposure to many BPA analogues leads to disrupted neuronal development, with immunohistochemistry and gene expression studies demonstrating decreased motor neuron lengths [80,87,91,92], alterations in neuron differentiation [79,93,94], neurogenesis [26,73,80,83,95], and neural maturation [93], in addition to a rise in brain degeneration [85] and neuron apoptosis [86]. Specific neurotransmitters including GABA, glutamine, glutamate, dopamine, serotonin, acetylcholine, and isotocin (oxytocin-family peptide) also show changes with perinatal analogue exposure [26,80,87,88]. Changes in specific ion channel expression in the brain have also been found [73]. Further evidence suggests alterations to the hypothalamus–pituitary–thyroid (HPT) [79,85,87] and HP–gonadal (HPG) [81,85,96,97] axes, circadian rhythms [91], and the visual system [90,95,98]. Similar to the behavioral results discussed above, BPAF seems to most severely alter genes and hormones compared to BPB, BPS, and BPF [81].

Regarding mechanisms of action, increases in oxidative stress are a consistent mechanism to which alterations to neurodevelopment and behavior through bisphenols, similar to results for BPA, are attributed [73,82,83,84,98].

**Table 2:** Neurodevelopmental and behavioral effects of non-BPA bisphenols in zebrafish.

EDC	Time of Exposure (hpf)	Dose	Findings	Reference
BHPF	4–144	300–4500 nM	↓ Larvae locomotion at 3000, 4500 nM ↓ Larvae recovery to stressful stimuli at 4500 nM in light/dark challenge ↑ CNS neuron differentiation at 300 and 500 nM ↓ CNS neuron differentiation at 750, 1500, 3000, 4500 nM ↑ Expression of *tshb*, *tg*, *nkx2.1*, *tshr*, *dio1*, *ugt1ab* at all doses; tuba 1b at all doses except 1500 nM; *crhb* in all doses except 750 nM; *dio2* at all doses except 750 nM and 1500 nM; *cfos* at doses above 750 nM; *gap43* at doses above 3000 nM (genes related to HPT axis)	Jin et al., 2021 [79]
BHPF	2–120	0.1, 10, 1000 nmol/L	↓ Expression of *pax2* expression in the spinal cord and mid-hindbrain at 10 and 1000 nM (genes related to brain morphology) ↑ Wake period during light periods at all doses, decreasing rest time ↓ Motor neuron length at all doses ↑ Expression of *hcrt* and *aanat2* (genes related to circadian rhythm) ↑ Expression of *hcrtr* at 0.1 and 10 nM; ↓ at 1000 nM (genes related to circadian rhythm)	Mi et al., 2019 [91]
BPAF	8–108	0.047, 0.47, 4.7 μM	↑ Nearest neighbor distance and inter-individual distance at 0.47 μM, indicating alterations in shoaling (social grouping) behavior	Bai et al., 2023 [73]
BPAF	2–144	0.1, 1 μM	↓ Larvae locomotion at 1 μM (0.1 μM not measured) ↑ AroB cells in POA at 1 μM and in NRP at both doses (related to neuroendocrine system) No effect on BrdU (mitosis marker) cells in POA and NRP	Coumailleau et al., 2020 [99]
BPAF	4–144	200 μg/L	↓ Embryo spontaneous movement ↓ Larvae locomotion speed ↓ motor neuron length at 36 and 72 hpf ↓ neurogenesis at 36 and 72 hpf ↓ glutamine, DOPA, dopamine, norepinephrine, tyramine, serotonin, 5-Hydroxy-L-tryptophan, Acetylcholine ↑ 3-methocytyramine, mornetanephrone, 5-hydroxyindoleacetic acid	Gu et al., 2022 [80]
BPAF	2–120	1, 100 μg/L	↓ Larvae locomotion at 100 μg/L ↑ GnRH terminal nerve and hypothalamic neurons with 100 μg/L dose ↑ Expression of *ren*, *pth*, *gh* at 1 μg/L; *kiss1*, *gnrh3*, *fshβ*, *lhβ*, *anp*, *ren*, *pth1*, *gh*, *prl* at 100 μg/L (genes related to reproductive neuroendocrinology) No effect on expression of *kiss2* at either dose	Qiu et al., 2021 [81]
BPAF	2–120	5, 50, 500 μg/L	↓ Larvae locomotion at all doses ↑ Oxidative stress at 500 μg/L	Rao et al., 2022 [82]
BPAF	35,186	200 μg/L	↓Larvae locomotion ↓ AChE activity ↓ Neurogenesis ↓ Expression of *elavl3*, *zn5*, *α-tubulin*, *syn2a*, and *mbp (*genes related to neurogenesis) ↑ Expression of *gap43* (gene related to neurogenesis) ↑ Oxidative stress ↑ Apoptosis in brain	Yang et al., 2023 [83]
BPAP	8–108	0.041, 0.41, 4.1 μM	↑ Nearest neighbor distance at 0.041 μM and inter-individual distance at 0.041 and 0.41 μM, indicating alterations in shoaling (social grouping) behavior	Bai et al., 2023 [73]
BPB	8–108	0.1, 1, 10 μM	↑ Nearest neighbor distance at all doses (no effect on inter-individual distance), indicating alterations in shoaling (social grouping) behavior	Bai et al., 2023 [73]
BPB	2–120	1, 100 μg/L	↓ Larvae locomotion at 100 μg/L ↑ GnRH brain neurons with 100 μg/L ↑ Expression of *kiss1*, *anp*, *ren*, *pth1*, *gh* at 100 μg/L (genes related to reproductive neuroendocrinology) No effect on expression of *kiss2*, *gnrh3*, *fshβ*, or *lhβ*, *prl* (genes related to reproductive neuroendocrinology)	Qiu et al., 2021 [81]
BPB	0–144	10, 100, 1000 μg/L	↓ Larvae locomotion at 100 and 1000 μg/L ↓ Embryo spontaneous movements at 100 and 1000 μg/L ↑ Oxidative stress at 100 and 1000 μg/L	Wang et al., 2023 [100]
BPB	0–144	1–1000 μg/L	↓ Larvae locomotion at 10–1000 μg/L ↑ Hypothalamus and mesencephalon degeneration at 100 and 1000 μg/L, with no change in other brain regions ↑ T3 hormone and T3/T4 hormone ratio at 10–1000 μg/L ↓ T4 hormone at 1000 μg/L ↑ Expression of *dio1*, *dio2* and *trhr1* at 1–1000 μg/L; *tg*, *ttr*, *thrα* at 10–1000 μg/L; *thrβ* at 100–1000 μg/L (genes related to the HPG axis) ↓ Expression of *tshβ* and *trh* at 1–1000 μg/L, (genes related to the HPG axis) ↓ Expression of *α1-tubulin, myelin basic protein*, *syn2a*, *elavl3*, *zn5* at 1–1000 μg/L; *gap43* at 100–1000 μg/L (genes related to neurodevelopment)	Yang et al., 2021 [85]
BPC	8–108	0.049, 0.49, 4.9 μM	↑ Nearest neighbor distance at all doses and inter-individual distance at 0.49 and 4.9 μM, indicating alterations in shoaling (social grouping) behavior	Bai et al., 2023 [73]
BPC	0–120	4.25, 8.5, 17 μg/L	↑ Larvae locomotion at all doses ↑ Time spent in well center at 8.5 μg/L	Blanc-Legendre et al., 2023 [74]
BPF	8–108	0.3, 3, 30 μM	↑ Embryo spontaneous movement at 3 μM ↑ Nearest neighbor distance at 0.3 and 3 μM and inter-individual distance at 3 μM, indicating alterations in shoaling (social grouping) behavior ↑ Locomotion in the dark period at all doses and ↓ in light period at 0.3 μM at 5 dpf ↓ Locomotion in light-dark exploration test at 3 μM and ↑ time in light at 3 and 30 μM at 10 dpf ↓ Time in social contact at 0.3 and 3 μM, and ↑ number of contacts at 30 μM ↑ Intraocular distance (ID) at 3 and 30 μM, lower jaw length (LJL), and Ceratohyal cartilage length (CCL) at all doses, indicating macrocephaly ↑ Neurogenesis at 3 μM (other doses not measured) ↑ Expression of *kctd12*, *kctd13*, *kcnab1a*, *kcnh4a*, *kcnj6* (K+ ion channel), *ryr1a*, *ryr1b*, *ryr3* (calcium ion channel) at 3 μM (other doses not measured) ↓ Expression of *chrna1* (AChr receptor) and *tnnc-2* (calcium ion channel) at 3 μM (other doses not measured) No change in expression of *kctd4* (K+ ion channel), *scn4bb* (sodium ion channel), *ryr2*, *tnnc-1* (calcium ion channel) at 3 μM (other doses not measured)	Bai et al., 2023 [73]
BPF	2–144	0.1, 1 μM	No effect on larvae locomotion ↑AroB cells in POA at 1uM and in NRP at both doses (related to neuroendocrine disruption) No effect on BrdU (mitosis marker) cells in POA and NRP	Coumailleau et al., 2020 [99]
BPF	4–144	200 μg/L	↓ Embryo spontaneous movement ↓ Larvae locomotion ↓ Neurogenesis at 36 and 72 hpf ↓ Motor neuron length at 36 hpf ↓ Glutamine, dopamine, norepinephrine, and serotonin ↑ DOPA, normetanephrine, 5-hydroxyindoleacetic acid, and Acetylcholine	Gu et al., 2022 [80]
BPF	0–72/144	7, 70, 700 μg/L	↓ Larvae locomotion at 70 and 700 μg/L administered until 6 dpf (not measured at 3 dpf) ↑ Apoptosis in larvae brain at 70 and 700 μg/L doses until 3 dpf (not measured at 6 dpf) ↓ Larvae expression of *α1-tubulin*, *Syn2a* at 700 μg/L, *elavl3*, *mbp*, and *gfap* at 70 and 700 μg/L after administering until 3 dpf. ↓ in all these genes at 70 and 700 μg/L when administered until 6 dpf (genes related to neurodevelopment) ↑ Aberrant brain nuclei arrangement in larvae	Gu et al., 2020 [86]
BPF	0–144	2, 20, 200 μg/L	↓ Embryo spontaneous movement at 20 and 200 μg/L ↓ Larvae locomotion at 2 and 20 μg/L (200 μg/L not) ↓ Expression of *Ngn1*, *Elavl3*, *mbp*, at 20 μg/L and *Nrd* at both 2 and 20 μg/L (200 μg/L not) (genes important for neuronal differentiation and development) ↑ Expression *syn2α*, *gfap*, and *gap43* at 2, and 20 μg/L and *α1-tubulin* at 20 μg/L (200 μg/L not measured) (genes related to neural maturation and regeneration)	Gu et al., 2022 [93]
BPF	4–120	5, 10 mg/L	↓ Larvae locomotion at all doses ↑ Time in center ↑ Delayed response to stimuli at 10 mg/L ↓ Motor neuron length and axonal branching at 10 mg/L ↓ Oligodendrocytes and length of myelin sheath at 10 mg/L ↓ TH, catechol-O-methyltransferase (COMT), dopamine beta-hydroxylase (DBH), allopregnanolone, testosterone at 10 mg/L (5 mg/L not measured) ↑ Progesterone at 10 mg/L (5 mg/L not measured)	Kim et al., 2023 [87]
BPF	2–96	0.0005, 0.5, 5.0 mg/L	↓ Embryo spontaneous movement at 0.5 and 5 mg/L ↓ Motor neuron length at 0.5 and 5 mg/L ↓ Expression of *socs3a*, *fosb*, and *nlgn2b*, at 0.0005 and 0.5 mg/L (5 mg/L not measured) (genes related to neurodevelopment)	Mu et al., 2019 [92]
BPF	2–120	1, 100 μg/L	No effect on larvae locomotion at all doses ↑ GnRH hypothalamic neurons with 100 μg/L No effect on GnRH terminal nerve neurons with 100 μg/L ↑ Expression of *kiss2*, *anp*, *ren*, *gh* at 1 μg/L; *lhβ*, *anp*, *ren*, *pth1* at 100 μg/L (genes related to reproductive neuroendocrinology) No effect on expression of *kiss1*, *gnrh3*, *fshβ*, and *prl*	Qiu et al., 2021 [81]
BPF	2–72	0.25, 0.5, 1 μM	↓ Size of GnRH brain neurons at 2 dpf for 0.25 μM and at 3 dpf for 0.5 and 1 μM	Weiler and Ramakrishnan 2019 [97]
BPS	8–108	1, 10, 100 μM	↑ Nearest neighbor distance at 10 μM and inter-individual distance at 10 and 100 μM, indicating alterations in shoaling (social grouping) behavior ↑ Locomotion in the dark period and light period at all doses at 5 dpf ↓ Locomotion in light-dark exploration test and ↑ time in light at all doses at 10 dpf ↓ Time in social contact and number of contacts at all doses ↑ Intraocular distance (ID), lower jaw length (LJL), and ceratohyal cartilage length (CCL) at 1 and 100 μM, indicating macrocephaly ↑ Neurogenesis at 1 μM (other doses not measured) ↑ Expression of *kctd4*, *kctd12*, *kctd13*, *kcnab1a*, *kcnh4a*, *kcnj6* (K+ ion channel), *ryr2*, *ryr3* (calcium ion channel), *scn4bb* (sodium ion channel) at 3 μM (other doses not measured) ↓ Expression of *chrna1* (AChr receptor) and *tnnc-2* (calcium ion channel) at 1 μM (other doses not measured) No change in expression of *ryr1a*, *ryr1b*, *tnnc-1* (calcium ion channel) at 3 μM (other doses not measured)	Bai et al., 2023 [73]
BPS	2–144	0.03, 0.3, 3.0 mg/L	↓ Larvae locomotion at all doses No changes in apoptosis at all doses ↓ Expression of *α1-tubulin* and *gap43* at 0.3 and 3 mg/L; *elavl3*, *mbp*, *syn2a*, and *gap43* at 3 mg/L, indicating disruption in neurodevelopment ↑ Retinal and optic nerve disruption at all doses	Gu et al., 2019 [95]
BPS	4–144	200 μg/L	No effect on larvae locomotion ↓ Neurogenesis at 36 hpf but not 72 hpf ↓ Motor neuron length at 36 hpf but not 72 hpf ↓ Norepinephrine, 5-hydroxy-L-tryptophan ↑ 3-methoxytyramine, tyramine	Gu et al., 2022 [80]
BPS	4–24/48/72/96/120	0.01–1 μM	↑ Larvae locomotion at 1 μM ↑ Expression of *elavl3* at 0.03 μM and 1 μM at 120 hpf; *ngn1* at all doses at 120 hpf and all but 0.01 μM at 48 hpf; *gfap* (genes related to neurodevelopment)	Gyimah et al., 2021 [94]
BPS	0–120	0.0068 μM	↑ Larvae locomotion ↑ Hypothalamic neurogenesis	Kinch et al., 2015 [101]
BPS	2–120	0.001, 0.01, 0.1 μM	↑ Time and distance in well peripheral zone at 0.001 μM ↓ Time in “social” zone at 0.1 μM ↓ Exploration of novel object in memory task at 0.01 and 0.1 μM ↑ Expression of *esr1*, *esr2a*, *it*, *slc12a5a*, *slc12a5b*, *slc12a2* at 0.001 μM (genes related to brain signaling pathways) ↓ Expression of *esr2b* at 0.01 and 0.1; *it* and *slc12a2* at 0.1 μM (genes related to brain signaling pathways) ↑ Expression of *gad1b*, *slc32a1*, *slc6a1a*, *gabra1* at 0.001 μM; *slc17a7a*, *grla1a*, *grin1a* at 0.1 μM (genes associated with GABA and glutamate signaling) ↓ Expression of *slc6a1a* and *gabra1* at 0.1 μM; *grabra2* at 0.01 and 0.1 μM (genes related to brain signaling pathways) ↑ Brain Isotocin at 0.01 μM	Naderi et al., 2022 [88]
BPS	2–25/120	0.1–1000 μg/L	↑ HYPO-GnRH3 neurons at 25 hpf with 100 μL/L (other doses not measured) No changes in TN-GnRH3 neurons at 25 hpf with 100 μL/L ↑ Expression of *kiss1*, *gnrh3*, *er-α* at 100 μL/L at 25 hpf with 100 μL/L (genes related to reproductive neuroendocrinology) No effect on *kiss2*, *kiss2r*, *fshβ*, *erβ*, and *sv2* expression at 25 hpf with 100 μL/L	Qiu et al., 2016 [96]
BPS	2–120	1, 100 μg/L	No effect on larvae locomotion at either dose ↑ GnRH hypothalamic neurons with 100 μg/L No effect on terminal nerve GnRH neurons at 100 μg/L ↑ Expression of *lhβ*, *anp*, *prl* at 1 μg/L and *kiss1*, *kiss2*, *lhβ*, *anp*, *ren*, *pth1*, *gh* at 100 μg/L (genes involved in reproductive neuroendocrinology) No effect on *gnrh3*, *fshβ* at all doses	Qiu et al., 2021 [81]
BPS	2–120	1, 10, 100 μg/L	↑ Development of optic nerve at 10 μg/L and retinal ganglion cells, hypothalamic neurons, and motor neurons at 10 and 100 μg/L ↓ Cone synapses at 10 and 100 μg/L ↑ Altered mosaic patterning of cones at 10 and 100 μg/L ↑ Oxidative stress-related genes in cones	Qiu et al., 2023 [98]
BPS	2–120	1, 10, 100 μg/L	↓ Yolk lipid supply and LCFA precursors which allow for brain development at all doses ↓ Brain lipid levels at 120 hpf at all doses	Wang et al., 2023 [102]
BPS	2–120	4, 400 nM	↓ Retinal thickness at all layers at all doses ↓ Light-seeking behavior at 400 nM ↓ Phototransduction genes at 400 nM	Wei et al., 2023 [90]
BPS-MPE	0–120	185, 570, 1140 μg/L	No effect on larvae locomotion for any of the doses ↓ Time spent in well center, indicating lower anxiety, at 570 and 1140 μg/L	Blanc-Legendre et al., 2023 [74]
DM-BPA	0–120	135, 370, 540 μg/L	↓ Larvae locomotion at 540 μg/L No difference in time spent in well center for any dose	Blanc-Legendre et al., 2023 [74]
HPP	8–108	0.061, 0.61, 6.1 μM	↑ Nearest neighbor distance and inter-individual distance at 0.61 μM, indicating alterations in shoaling (social grouping) behavior	Bai et al., 2023 [73]
TMBPF	4–144	0.25–8 mg/L	↓ Embryo spontaneous movement at all doses ↓ Larvae locomotion at 0.5 mg/L and above ↓ Neurogenesis in larvae brain at 0.5 mg/L and above ↓ Larvae motor-neuron length at 0.5 mg/L and above ↓ Dopamine neuron development at all doses ↓ Expression of *syn2a* and *gafp* expression at 0.5 mg/L and above (genes related to neurodevelopment) ↑ Expression of *th1* and *th2* at 0.24 mg/L and above (genes related to neurodevelopment)	Liang et al., 2023 [26]
BPAF, BPB, BPF, BPS, BPS-MAE, TCBPA	0–120	^1^	No effect on larvae locomotion or time spent in well center	Blanc-Legendre et al., 2023 [74]
BPE, BPP, BPZ	8–108	^2^	No effect on shoaling (social grouping) behavior	Bai et al., 2023 [73]
BPS, BPAP	2–144	0.1, 1 μM	No effect on larvae locomotion No effect on AroB (neuroendocrine marker) and BrdU (mitosis marker) cells in POA and NRP	Coumailleau et al., 2020 [99]

All effects were statistically significant at *p* < 0.05. ↓ refers to a decrease while ↑ refers to an increase in the EDC group(s) compared to a control. ^1^ μg/L: 67.5–270 (BPAF), 150–600 (BPB), 100–400 (BPF), 15,750–63,000 (BPS), 87.5–350 (BPS-MAE), 62.5–250 (TCBPA). ^2^ μM: 0.31–31 (BPE), 1–100 (BPP), 0.068–6.8 (BPZ).

### 4.2. Bisphenols and Rodents

Research assessing the influence of early-life single-BPA-analogue exposure in rodents is limited to BPAF, BPAP, BPF, and BPS. All four of these chemicals demonstrate adverse neurodevelopmental and behavioral effects (Table 3).

Early-life BPAF, BPAP, BPF, and BPS exposure resulted in decreased time or number of entries in the center of the open field and open arms of the elevated plus maze in males [103,104,105,106,107] and females [104,105,106,108,109]. BPS produced the least consistent results of the four bisphenols, with multiple studies finding no effect on behavior in these anxiety-related tests [107,109,110,111,112]. Tests of depression-like behaviors, including the novelty-suppressed feeding test (food-deprived animal chooses between a dark chamber or light chamber with food), tail suspension test, forced swim test, and sucrose preference test indicate effects in both sexes, but primarily males, exposed to BPAF [103,104,108]. In one study, BPF exposure increased immobility in female mice in the forced swim test [106], while a study with 1/200th of the dosage found no effect in the tail suspension and forced swim tests [111]. No effects of early BPS exposure were observed on depression-like behaviors [107,111]. Multiple studies also assessed the effect of early BPA analogue exposure on learning and memory. BPAF and BPAP in both sexes affected long-term and spatial memory [103,104,105,113]. Meanwhile, a decrease in olfactory short-term memory was found with early BPF exposure [114], another study at a similar dose found no impact on cognition and memory [111]. Early BPS exposure produced no identifiable effects on memory in another study [111]. Social behavior alterations were also present after perinatal exposure to BPAF [104], BPAP [105], BPF [111], and BPS [111,112], in addition to altered maternal behavior after BPS exposure.

All of the above bisphenol analogues have been shown to influence neurodevelopment through decreasing synapse and spine densities [113]. Alterations in gene expression and the transcriptome related to neuron differentiation, synaptic signaling, and organization were also observed [104,105,114]. Studies also demonstrate the effects of specific neurotransmitters, including dopamine and serotonin [109,115], as well as the disruption of hormone systems important for neurodevelopment, such as the estrogen system [110] and 5α-reductase, an enzyme important for hormone metabolism [115]. Evidence suggests microglia and oxidative stress may be drivers in these neurodevelopmental disruptions caused by bisphenols [105,113]. 

**Table 3:** Neurodevelopmental and behavioral effects of non-BPA bisphenols in rodents.

EDC	Animal	Time of Exposure	Dose	Findings	Reference
BPAF	Mice	GD 0.5–18.5	0.4, 4 mg/kg	↓ Time in center of open field at all doses in females ↑ Latency to feeding at all doses, total intake at 4 mg/kg, and ↓ total intake at 0.4 mg/kg in females in novelty-suppressed feeding test No effect on the latency to feeding, but ↓ total intake in males at all doses in novelty-suppressed feeding test ↑ Immobility time at all doses but no effect on latency to immobility in females in the tail suspension test ↓ Immobility time at 4 mg/kg and latency to immobility at 0.4 mg/kg in males in tail suspension test ↑ Floating time at 4 mg/kg for females in forced swim test. No effect of floating time in males or latency to floating in males or females. ↓ Sucrose preference for females in 0.4 mg/kg test No effect on short-term or long-term memory in the novel object recognition test	Gong et al., 2022 [108]
BPAF	Mice	GD 1–19	0.4, 4 mg/kg	↓ Time in center of open field at both doses in males No effect on locomotion in open field ↑ Latency to feeding at both doses in males and ↓ latency to feeding at 0.4 mg/kg in females in the novelty-suppressed feeding test ↓ Sucrose preference in males at 0.4 mg/kg ↑ Immobility in tail suspension test in males at both doses ↑ Floating time in forced swim test in females at 4 mg/kg ↓ Long-term memory at both doses for males and 0.4 mg/kg for females in novel object recognition test. No change seen in short-term memory. ↓ Freezing time in both long-term and short-term memory in contextual fear conditioning test in males at both doses	Gong et al., 2017 [103]
BPAF	Mice	GD 6–P 21	0.34, 3.4, 34 mg/kg	↓ Time spent in target quadrant of MWM in males at all doses, indicating impaired spatial memory ↓ Number of quadrant crossing of MWM in females at 34 mg/kg, indicating impaired spatial memory No effect on hippocampal neuronal damage ↓ Number of intersections in CA1 and DG neurons at all doses in males and only at 34 mg/kg in DG neurons in females ↓ Spinal density in dendrites at 3.4 and 34 mg/kg in males No effect on spinal density in females ↓ PSD-95 at 3.4 and 34 mg/kg and Synapsin-1 at all doses in males; PSD-95 at 34 mg/kg in females ↓ Hippocampal ERα in males at all doses and ERβ in females at all doses No effect on hippocampal ERβ in males and ERα in females ↑ Oxidative stress in male hippocampus (females not tested) No effect on brain weight	Zhang et al., 2021 [113]
BPAF	Mice	GD 7–P 0	0.4 mg/kg	↓ Entries/time in center and locomotion in open field for both males and females ↓ Open-arm time and locomotion in EPM for males ↑ Marble burying in MBT for males ↑ Latency to first immobility time and immobility in the tail suspension test for males ↓ Time with novel object in novel object recognition test for males ↓ Sociability for males ↑ Alterations in transcriptome related to synaptic signaling, organization, and structure, neurotransmitters, and neuron development for males (females not tested)	Wu et al., 2023 [104]
BPAP	Mice	GD 7–P 21	0.4 mg/kg	↓ Entries and velocity in center for both males and females distance traveled in center for males in the open-field test. No effect on time in center ↓ Time in open arms for females in the elevated plus maze ↑ Number of marbles buried for males and females in marble-burying test ↓ Time spent with novel objects in males but not females. ↓ Preference index for novel object in both males and females in novel object recognition test ↓ Time spent with mouse compared to empty cage and novel mouse compared to familiar mouse in three-chamber test in both males and females ↓ Surviving neurons in male and female CA1 and DG ↑ Alterations in transcriptome related to astrocytes, microglia, neurons, oligodendrocytes, and pathways associated with Parkinson’s and neurodegeneration. ↑ Expression of *C1qc*, *Ctss*, and *Iba1* (genes related to microglia) ↓ *Il1rapl1, Sgk3, Ncam2, Kirrel, Fkbp5* (genes related to neurodevelopment) ↑ Macrophages and activation of dendritic cells	Wu et al., 2023 [105]
BPF	Mice	GD 15–P 21	2, 200 µg/kg	↓ Short-term olfactory memory at 200 µg/kg No effect on serum TH levels ↓ Neurogenesis and corpus collosum thickness at 200 µg/kg No effect on oligodendrogenesis, oligodendrocyte differentiation, or myelination ↑ Alterations in transcriptome related to brain development, neuron fate development, neuron differentiation at both doses, in addition to myelination and oligodendrogenesis at 200 µg/kg No alterations in genes related to intracellular TH, important for brain development	Vancamp et al., 2023 [114]
BPF	Mice	GD 9.5–P 28	50 μg/kg	No effect on sociability ↓ Time spent with novel mouse in three-chamber test ↓ Sniffing of conspecific in open field No effect on anxiety as measured in open field and elevated plus maze No effect on depressive behavior as measured in the tail suspension test and the forced swim test No effect on locomotion and motor learning as measured in rotarod test No effect on cognition and memory as measured in novel object test	Moon et al., 2023 [111]
BPF	Rats	GD 12–P 21	10 μg/kg	↓ 5α-reductase type 3 but not type 1 or 2 mRNA ↑ Expression of *Cyp2d4*, *Htr4*, *Nr4a1* (dopamine and serotonin genes) ↓ *Htr1d*, *Pde4c*, *Adcy1*, *Ddc*, *Dbh*, *Adcy2*, *App*, *Htr1a*, *Comt*, *Syn2*, *Fos*, *Akt3*, *Akt1*, *Tph1*, *Adcy5*, *Adrb2*, *Bdnf* (dopamine and serotonin genes)	Castro et al., 2015 [115]
BPF	Mice	GD 11.5–18.5	10 mg/kg	No effect on locomotion ↓ Time in center of open field for females ↑ Time spent in closed arm of EPM for both females and males ↑ Time immobile in forced swim test for females	Ohtani et al., 2017 [106]
BPS	Mice (Female only)	GD 9–P 21	2, 200 µg/kg	No effect on the open field ↑ Infanticide, pup neglect, and improper pup care carried out by exposed female offspring at 2 μg/kg ↓ Time spent in nest at both doses ↑ Nest building at 200 μg/kg No effect on time spent grooming, nest size ↓ Latency for pup retrieval No effect on ERα in the MPOA and TH in the VTA	Catanese and Vandenberg, 2016 [110]
BPS	Mice	GD 9.5–P 28	50 μg/kg	No effect on sociability ↓ Time spent with novel mouse in three-chamber test ↓ Sniffing of conspecific in open field No effect on anxiety as measured in open field and elevated plus maze No effect on depressive behavior as measured in the tail suspension test and the forced swim test No effect on locomotion and motor learning as measured in rotarod test No effect on cognition and memory as measured in novel object test	Moon et al., 2023 [111]
BPS	Rats	GD 12–P 21	10 μg/kg	↓ 5α-reductase type 3 but not type 1 or 2 mRNA ↑ Expression of *Cyp2d4*, *Htr4*, *Nr4a1*, *Dusp1*, and *Pde4b* (dopamine and serotonin genes) ↓ *Adct2*, *Adrb2*, and *Tph1* (dopamine and serotonin genes)	Castro et al., 2015 [115]
BPS	Rats	GD 0–P 20	10, 50 μg/kg	No effect on open field ↓ Time and entries in open arms of males at both doses ↑ High-fat diet consumption at both doses in males and at 10 μg/kg in females No effect on high-sugar diet consumption	Da Silva et al., 2019 [107]
BPS	Mice	GD 0–P 28	4 μg/kg	↓ Open-arm time and ↑ locomotion in EPM in females but not males No effect on time spent in center of open field for males and females, ↓ latency to first entry into center for males ↑ Serotonin neurons in DRV and serotonin fractional area in DR and DRV in males and in serotonin fractional area in DRD in females (DR = Dorsal Raphe Nucleus, DRD = dorsal region of DR, DRV = ventral region of DR). No effects in the Median Raphe Nucleus.	Bonaldo et al., 2023 [116]
BPS	Mice	GD 9–P 20	2, 200 µg/kg	↓ Litters initiating nursing at 200 μg/kg	LaPlante et al., 2017 [117]
BPS	Mice	GD 8–P 21	0.2 mg/kg	No effect on open-field test ↓ Time spent with familiar mice and ↑ locomotion in social test	Kim et al., 2015 [112]

All effects were statistically significant at *p* < 0.05. ↓ refers to a decrease while ↑ refers to an increase in the EDC group(s) compared to a control.

### 4.3. Bisphenols and Humans

The literature on human studies is based on epidemiological and population studies relating exposure or body burden to outcomes. A wide variety of BPA analogues have been considered, although most bisphenol replacements have had levels of detection that were too low for further analysis [118]. Because of this, most studies to date have only assessed the relationship between perinatal and childhood exposure to BPAF, BPF, and BPS on measures of cognition and behavior, and results have varied, no doubt due to differences in the populations, methodology of behavioral testing, and other experimental factors (Table 4). While early BPAF exposure was associated with lower levels of social development in girls [119], no associations were found with IQ, scores of perceptual reasoning, verbal comprehension, gross and fine motor skills, adaptive skills, and language [119,120]. Early BPF exposure was linked to lower IQ, perceptual reasoning, and verbal comprehension in boys [120,121] and higher ADHD rating scores in both sexes (although higher in girls) [122]. Other studies found no association between BPF exposure and IQ and measures of infant neurodevelopment [123,124]. Early BPS exposure was correlated with increases in ADHD rating scores in both sexes [122], an increase in emotionally reactive behaviors in girls [125], and a decrease in psychomotor development in boys [123]. Multiple studies found no correlations with early BPS exposure and IQ, mental development, and other measures of cognition [118,119,120,121,123,124,126,127]. Again, the disparities in results can likely be attributed to the different characteristics of the populations studied.

## 5. PFAS Alternatives and Neurodevelopment

### 5.1. PFAS and Zebrafish

A wide array of PFAS alternatives cause behavioral and neurodevelopmental effects in zebrafish (Table 5). Commonly studied alternatives for the legacy chemicals PFOA, PFOS, and PFHxS include PFBS, PFHpA, 6:2 FTS, 6:2 FTSA, Gen-X, PFBA, PFHpS, and PFNA. Embryonic exposure to all of these chemicals, except 6:2 FST, caused alterations in larvae locomotion and photomotor (movement in response to light) response [129,130,131,132,133,134,135,136]. In terms of neurodevelopmental effects, studies showed that early-life GenX and PFHxA exposure caused an alteration in genes related to neuron differentiation and growth [129,136]. PFBA exposure led to an increase in the enrichment of pathways associated with neurological disorders and neurodevelopment [132]. 

Truong et al. compared 139 PFASs and assessed larvae and embryonic photomotor response, a measure of neurodevelopmental toxicity. Interestingly, they found that the average mass of the compound, number of fluorinated carbons, functional head groups, and physicochemical properties (e.g., vapor pressure) were not predictive of behavior. They did find that less volatile PFASs were associated with alterations in these neurodevelopmental tests, although the implications for non-aquatic models are unknown. Rericha et al. similarly tested 58 PFASs and found no conclusive association between chain length and neurotoxicity as well, with alterations to the behavior observed among a variety of chain lengths and subclasses. Gaballah et al., who explored the effects of chain length in five aliphatic sulfonic acid PFASs, found that although chain length was generally associated with neurotoxicity, exceptions exist, bringing into question the notion that smaller chain length PFASs are safer. 

### 5.2. PFAS and Rodents

Only limited studies have assessed the influence of new-generation PFAS on rodent neurodevelopment and behavior. In fact, we only found two studies that evaluated the effects of the perinatal administration of GenX on thyroid hormone during gestation, which is essential for the proper development of the brain (Table 6) [143,144]. Early GenX exposure altered placental thyroid hormone levels, potentially disrupting offspring neurodevelopment [143,144].

### 5.3. PFASs and Humans

Many PFASs measured in human subjects fall below the level of detection, making analysis difficult. PFASs with concentrations above the level of detection include 6:2Cl-PFESA, EtFOSAA, MeFOSAA, PFBS, PFDA, PFDeA, PFDoA, PFHpA, PFHpS, PFNA, and more (Table 7). PFBS, PFDeA, PFDoA, PFNA, and PFUnDA have demonstrated an inverse relationship with IQ or other measures of cognition [145,146,147,148,149,150]. Associations between early PFAS exposure and autism spectrum disorder (ASD) or deficits in social behavior were observed with 6:2 Cl-PFESA, PFBS, PFDA, PFDoA, and PFNA [146,151,152,153,154]. Positive correlations have also been seen with early PFAS exposure and ADHD, specifically with exposure to PFDA, PFNA, and PFUnDA [128,155]. Deficits in cognition, social behavior, and other behavioral alterations were found with a variety of PFASs from different subclasses and with different chain lengths, including short-chain PFASs such as PFBS. Concurrently, many studies found null correlations with the chemical discussed above and measures of cognition, ASD, ADHD, and more. Differences in findings may be a result of variations in levels of exposure, participant demographics, and the timing of exposures measured. 

It is surprising that some studies observed apparent beneficial relationships between PFAS exposure and neurodevelopment and behavior. Harris et al. found that early-life PFNA, EtFOSAA, and MeFOSAA exposure were associated with higher visual–motor scores. They hypothesized that PFASs at specific concentrations may be neuroprotective through their agonist activity on peroxisome proliferator-activated receptor gamma (PPAR-γ), leading to reduced inflammation [156,157,158]. Luo et al. also found that PFBS, PFDoA, and PFUnDA were positively associated with social–emotional and/or adaptive scores [146]. Skogheim et al. found positive correlations between PFDA, PFNA, PFHpS, and PFUnDA with verbal working memory. Enright et al. found that early exposure to PFNA, PFDeA, and PFUdA was associated with better visual attention [159]. Finally, PFNA was positively associated with visual–spatial and working memory, in addition to vocabulary scores [160,161,162]. While the seemingly beneficial effects of certain PFASs are notable, it is important to consider that these same chemicals can (and do) have adverse effects on other biological systems. 

**Table 7:** Associations with non-PFOA, -PFOS, and -PFHxS PFASs and neurodevelopment and behavior in humans.

EDC	Time EDC Measured	Concentration Measured	Findings	Reference
6:2Cl-PFESA	Birth	Median Serum (Cord): 2.05 μg/L	↓ Association with communication and gross-motor scores	Zhou et al., 2023 [152]
EtFOSAA	Gestation (<22 weeks) and 6.6–10.9 years	Range Plasma (ng/mL): Prenatal: <0.1–44.6 Childhood: NA	↑ Association with 0.8–1.1 ng/mL prenatal exposure and mid-childhood visual–motor score No association with vocabulary scores, verbal and nonverbal IQ, and visual–spatial perception and memory	Harris et al., 2018 [156]
EtFOSAA	Gestation (<22 weeks) and 6.6–10.9 years	Range plasma (ng/mL): Prenatal: <0.1–33.6 Childhood: NA	↓ Association with prenatal 0.8–1.1 ng/mL quartile and teacher-rated behavioral regulation and metacognition index problems	Harris et al., 2021 [163]
MeFOSAA	Gestation (<22 weeks) and 6.6–10.9 years	Range Plasma (ng/mL): Prenatal: 0.1–29.7 Childhood: NA	↑ Association with prenatal 1.3–1.9 ng/mL and scores in an assessment of visual–motor abilities and an assessment of vocabulary No association with verbal and nonverbal IQ or visual–spatial perception and memory	Harris et al., 2018 [156]
MeFOSAA	Gestation (<22 weeks) and 6.6–10.9 years	Range plasma (ng/mL): Prenatal: 0.1–29.7 Childhood: NA	↓ Association with prenatal 2–3.1 ng/mL quartile and parent-rated total difficulties and internalizing scores (emotional and peer problems)	Harris et al., 2021 [163]
PFBS	Gestation (13–16 weeks)	Range Plasma: 0.01–7 ng/mL	↑ Association with social–emotional and adaptive scores	Luo et al., 2022 [146]
PFBS	Birth	Range Serum (Cord): 0.01–0.98 ng/mL	↓ Association with gross motor and adaptive skills in boys ↓ Association with social score ↓ Association with TSH and FT4 hormones No association with fine motor and language domain scores	Yao et al., 2022 [151]
PFBS	Gestation (9–16 weeks)	Median Serum: 0.05 ng/mL	↓ Association with IQ	Wang et al., 2023 [145]
PFDA	Birth	Median Serum (Cord): 0.24 μg/L	↓ Association with communication scores	Zhou et al., 2023 [152]
PFDA	2 and 4 years	Range Serum (ng/mL): 2 years: 0.07–1.25 4 years: 0.06–1.27	↑ Association with lower exposure at 2 years old and ADHD rating score	Kim et al., 2023 [164]
PFDA	Gestation (12–16 weeks)	Mean Plasma: 2.1 ng/mL	↑ Association with personal–social skills problems in girls No association with gross and fine motor skills and problem-solving skills	Niu et al., 2019 [153]
PFDA	Gestation (10–40 weeks)	Range Plasma: 0.02–4.02 ng/mL	↑ Association with problem behaviors ↑ Association with hyperactivity score	Hoyer et al., 2018 [165]
PFDA	Gestation (17 weeks)	Range Plasma: 0.05–1.77 ng/mL	↑ Association with verbal working memory in boys No association with ADHD symptoms, language skills, or IQ	Skogheim et al., 2021 [166]
PFDA	Gestation (32 weeks), 5 and 7 years	Range Serum (ug/L): Gestation: 0.03–0.98 5 years: 0.05–1.2 7 years: 0.07–2.02	↑ Association with 5-year exposure and total behavioral development scores, externalizing problems, hyperactivity/inattention, and conduct problems	Oulhote et al., 2016 [167]
PFDA	Gestation (<22 weeks) and 6.6–10.9 years	Range Plasma (ng/mL): Prenatal: NA Childhood: <0.1–1.9	↑ Association with childhood 0.5–1.9 ng/mL quartile and parent-rated total difficulties, internalizing scores (emotional and peer problems), and externalizing scores (hyperactivity and conduct problems)	Harris et al., 2021 [163]
PFDA	Gestation (18 weeks)	Range Plasma: 0.19–0.24 ng/mL	↓ Association with ADHD No association with ASD	Skogheim et al., 2021 [166]
PFDA	Gestation (~37 weeks)	Range Serum: <0.01–5.74 ng/mL	↓ Association with gross motor function score No association with fine motor function, communication, problem-solving ability, and personal–social skills	Li et al., 2023 [168]
PFDA	9–11 years old	Mean Blood: 0.26 ng/mL	↓ Association with response inhibition	Gump et al., 2012 [169]
PFDeA	Gestation (12–28 weeks)	Mean Serum: 0.08 ng/mL	↑ Association with attention No association with information processing speed and visual recognition memory	Enright et al., 2023 [159]
PFDeA	Gestation (13–16 weeks)	Range Plasma: 0.03–27.8 ng/mL	↓ Association with cognition, language, and motor scores	Luo et al., 2022 [146]
PFDoA	Gestation (12–16 weeks)	Mean Plasma: 0.1 ng/mL	↑ Association with personal–social skills problems in girls No association with gross and fine motor skills and problem-solving skills	Niu et al., 2019 [153]
PFDoA	Gestation (13–16 weeks)	Range Plasma: 0.04–2.9 ng/mL	↓ Association with cognition and language scores ↑ Association with adaptive scores	Luo et al., 2022 [146]
PFDoDA	Gestation (~37 weeks)	Range Serum: <0.01–2.01 ng/mL	↓ Association with problem-solving ability No association with communication, gross and fine motor function, and personal–social skills	Li et al., 2023 [168]
PFHpA	Gestation (10–40 weeks)	Range Plasma: 0.003–0.42 ng/mL	↑ Association with hyperactivity score No association with problem behaviors	Hoyer et al., 2018 [165]
PFHpA	Gestation (13–16 weeks)	Range Plasma: 0.01–2.49 ng/mL	↓ Association with language and motor scores	Luo et al., 2022 [146]
PFHpS	Gestation (18 weeks)	Range Plasma: 0.17–0.23 ng/mL	↑ Association with ASD in girls No association with ADHD	Skogheim et al., 2021 [166]
PFHpS	Gestation (17 weeks)	Range Plasma: 0.05–0.62 ng/mL	↓ Association with nonverbal working memory No association with ADHD symptoms, language skills, or IQ	Skogheim et al., 2020 [170]
PFNA	Birth	Median Serum (Cord): 0.34 μg/L	↓ Association with communication scores	Zhou et al., 2023 [152]
PFNA	Gestation (13–19 weeks), 3 years, and 8 years	Range Serum (ng/mL): Prenatal: 0.1–2.9 3 years: 0.5–41.7 8 years: 0.1–5.2	↑ Association with 3- and 8-year-old exposure and completion time of a visual spatial abilities test No association with spatial reference memory, errors of omission (inattention), and reaction time in an attention and impulsivity test	Vuong et al., 2018 [160]
PFNA	Gestation	Range Serum: 0.2–1 ng/mL	↑ Association with ASD	Oh et al., 2021 [154]
PFNA	Gestation (11–15 weeks)	Median Plasma: 0.7 ng/ml	↑ Association with cognitive development ↓ Association with working memory scores	Carrizosa et al., 2021 [147]
PFNA	Gestation (10–30 weeks)	Median Serum: 0.9 ng/mL	↑ Association with DNA methylation sites near genes DPAGT1, SLC6A2, and TMEM56 (related to neuromuscular transmission, ADHD, depression, and bipolar disorder)	Liu et al., 2022 [171]
PFNA	Gestation (13–19 weeks, 26 weeks, at delivery)	Mean Serum: 0.90 ng/mL	↑ Association with externalizing problems, including hyperactivity; Behavior Symptoms Index ↑ Association with ADHD symptoms and criteria	Vuong et al., 2021 [155]
PFNA	Gestation (6–26 weeks)	Median Plasma: 0.46 ng/mL	↑ Association with IQ	Liew et al., 2018 [172]
PFNA	2 and 4 years	Range Serum (ng/mL): 2 years: 0.15–17.4 4 years: 0.13–7.56	↑ Association with lower exposure at 2 years old and ADHD rating score	Kim et al., 2023 [164]
PFNA	Gestation (12–16 weeks)	Mean Plasma: 1.8 ng/mL	↑ Association with personal–social skills problems in girls No association with gross and fine motor skills and problem-solving skills	Niu et al., 2019 [153]
PFNA	Gestation (13–19 weeks), 3 years, and 8 years	Median Serum (ng/mL): Prenatal: 0.9 3 years: 1.2 8 years: 0.7	↑ Association with prenatal exposure and childhood exposure and reading scores	Zhang et al., 2018 [173]
PFNA	Gestation (13 weeks–birth), 3 years, 8 years	Mean Serum (ng/mL): Gestation: 0.9 8 years: 0.8	↑ Association with prenatal exposure and working memory ↑ Association with prenatal and child exposure and processing speed and verbal comprehension ↑ Association with child exposure and IQ and perceptual reasoning	Vuong et al., 2019 [161]
PFNA	Gestation (10–40 weeks)	Range Plasma: 0.14–5.71 ng/mL	↑ Association with problem behaviors ↑ Association with hyperactivity score	Hoyer et al., 2018 [165]
PFNA	Gestation (17 weeks)	Range Plasma: 0.06–5.32 ng/mL	↑ Association with verbal working memory in boys No association with ADHD symptoms, language skills, or IQ	Skogheim et al., 2020 [170]
PFNA	Gestation (<22 weeks) and 6.6–10.9 years	Range Plasma (ng/mL): Prenatal: <0.1–6.0 Childhood: <0.1–25.7	↑ Association with 1–6 ng/mL prenatal exposure and visual–spatial perception and memory No association with prenatal exposure and visual–motor scores, vocabulary, and verbal and nonverbal IQ. No association with childhood exposure and visual–motor scores, vocabulary, IQ, and visual–spatial perception and memory.	Harris et al., 2018 [156]
PFNA	Gestation (32 weeks), 5 and 7 years	Range Serum (ug/L): Gestation: 0.12–1.93 5 years: 0.39–6.16 7 years: 0.47–9.49	↑ Association with 5-year exposure and total behavioral development scores, externalizing problems, hyperactivity/inattention, and conduct problems ↑ Association with 7-year exposure and total behavioral development scores in girls; ↓ association with 7-year exposure and total behavioral development scores in boys	Oulhote et al., 2016 [167]
PFNA	Gestation (12–28 weeks)	Mean Serum: 0.28 ng/mL	↑ Association with attention No association with information processing speed and visual recognition memory	Enright et al., 2023 [159]
PFNA	Gestation (<22 weeks) and 6.6–10.9 years	Range plasma (ng/mL): Prenatal: <0.1–6.0 Childhood:<0.1–25.7	↑ Association with childhood 2.4–25.7 ng/mL and parent-rated total difficulties, internalizing (emotional and peer problems), and externalizing scores (hyperactivity and conduct problems) ↑ Association with 1.1–1.5 ng/mL childhood quartiles and teacher-rated total difficulties and the 1.6–2.3 ng/mL quartile and externalizing score	Harris et al., 2021 [163]
PFNA	Gestation (~16 weeks)	Median Serum: 3 years: 1.9 ng/mL 8 years: 1.2 ng/mL	↓ Association with 8-year exposure and metacognition scores, including initiation, planning, and organization of materials; association with 8-year exposure; and executive function ↑ Association with risk metacognitive impairments	Vuong et al., 2018 [174]
PFNA	Gestation (13–16 weeks)	Range Plasma: 0.05–16.97 ng/mL	↓ Association with cognition, language, and motor scores	Luo et al., 2022 [146]
PFNA	Gestation (8–16 weeks) and 18 months old	Median Serum (ng/mL): Prenatal: 0.65 Childhood: 0.57	↓ Association with prenatal exposure and IQ ↑ Association with childhood exposure and IQ	Beck et al., 2023 [175]
PFNA	At delivery	Range Cord blood: 0–10.3 ng/mL	↓ Association with psychomotor development index and verbal IQ	Spratlen et al., 2020 [150]
PFNA	Gestation	Mean Serum (ng/mL): 0.48	↓ Association with receptive language scores	Oh et al., 2021 [176]
PFNA	9–11 years old	Mean Blood: 0.82 ng/mL	↓ Association with response inhibition	Gump et al., 2011 [169]
PFNA	Gestation (28–40 weeks)	Median Serum: ~1.44–1.58 ng/mL	↓ Association with verbal, performance, and total IQ	Wang et al., 2015 [149]
PFNA	Gestation (~15 weeks)	Median Serum: 0.5 ng/mL	↓ Association with vocabulary score when mothers were <25 years old ↑ Association with vocabulary score when mothers were >30 years old	Jeddy et al., 2017 [162]
PFNA, PFDA, PFUA, Me-FOSAA, PFDOA, Et-FOSAA	Maternal serum collected at 2–5 years to calculate maternal prenatal serum	Range Reconstructed maternal prenatal serum: <0.49–>0.91 ng/mL (see paper for details)	No association with ASD	Shin et al., 2020 [177]
PFOSA	9–11 years old	Mean Blood: 0.75 ng/mL	↓ Association with response inhibition	Gump et al., 2011 [169]
PFTrDA	Gestation (22–15 weeks) and at delivery	Median Serum (ug/mL): Prenatal: 0.24 Birth (Cord Serum): 0.47	↓ Association with T3 and T4 No association with TSH	Kim et al., 2011 [178]
PFUdA	Gestation (12–16 weeks)	Mean Plasma: 1.6 ng/mL	↑ Association with personal–social skills problems in girls No association with gross and fine motor skills and problem-solving skills	Niu et al., 2019 [153]
PFUdA	Gestation (12–28 weeks)	Mean Serum: 0.04 ng/mL	↑ Association with attention No association with information processing speed and visual recognition memory	Enright et al., 2023 [159]
PFUnDA	2 and 4 years	Range Serum (ng/mL): 2 years: 0.08–1.8 4 years: 0.23–5.98	↑ Association with lower exposure at 2 years old and ADHD rating score	Kim et al., 2023 [164]
PFUnDA	Gestation (17 weeks)	Range Plasma: 0.05–1.46 ng/mL	↑ Association with verbal working memory in boys No association with ADHD symptoms, language skills, or IQ	Skogheim et al., 2020 [170]
PFUnDA	Gestation (18 weeks)	Range Plasma: 0.23–0.32 ng/mL	↓ Association with ADHD No association with ASD	Skogheim et al., 2021 [166]
PFUnDA	Gestation (13–16 weeks)	Range Plasma: 0.04–24.46 ng/mL	↓ Association with cognition, language, and motor scores ↑ Association with adaptive scores	Luo et al., 2022 [146]
PFUnDA	Gestation (~37 weeks)	Range Serum: <0.01–3.56 ng/mL	↓ Association with gross motor function score No association with communication, problem-solving ability, fine motor function, and personal–social skills	Li et al., 2023 [168]
PFUnDA	Gestation (28–40 weeks)	Median Serum: ~3.13–3.42 ng/mL	↓ Association with performance IQ	Wang et al., 2015 [149]
6:2 Cl-PFESA, 8:2 Cl-PFESA, PFBA, PFNA, PFTrDA	Gestation (~37 weeks)	Range Serum (ng/mL): <0.01–8.69 (6:2 Cl-PFESA), <0.01–0.23 (8:2 Cl-PFESA), <0.01–15.36 (PFBA), <0.01–2.68 (PFNA), <0.01–3.51 (PFTrDA)	No association with communication, problem-solving ability, gross and fine motor function, and personal–social skills	Li et al., 2023 [168]
FOSA	Gestation (<22 weeks) and 6.6–10.9 years	Range Plasma (ng/mL): Prenatal: NA Childhood plasma: <0.1–0.5	No associations found with childhood exposure and parent and teacher-rated strengths, difficulties, and executive function (including emotional and conduct problems)	Harris et al., 2021 [163]
Me-PFOSA-AcOH	Gestation (12–28 weeks)	Mean Serum: 0.04 ng/mL	No association with attention, information processing speed, and visual recognition memory	Enright et al., 2023 [159]
PFDA	Birth	Range Serum (Cord): 0.01–0.87 ng/mL	No association with gross and fine motor, adaptive, language, and social domain scores No association with thyroid hormone levels	Yao et al., 2022 [151]
PFDA, PFUnDA, PFDoDA, MeFOSAA, EtFOSAA	Gestation	Mean Serum: 0.1–0.19 ng/mL (see paper for details)	No association with scores of cognitive development	Oh et al., 2021 [176]
PFDA, Me-PFOSA-AcOH	Gestation (~16 weeks)	Median Serum (ng/mL): 3 years: 0.2–1.2 8 years: 0.2–0.7 (see paper for details)	No association with executive function or risk metacognitive impairments	Vuong et al., 2018 [174]
PFDeA	Gestation (<22 weeks) and 6.6–10.9 years	Range Plasma (ng/mL): Prenatal: <0.1–3.0 Childhood: NA	No association with visual–motor scores, vocabulary, verbal and nonverbal IQ, and visual–spatial perception and memory	Harris et al., 2018 [156]
PFDoA	Birth	Range Serum (Cord): 0.09–0.76 ng/mL	No association with gross and fine motor, adaptive, language, and social domain scores No association with thyroid hormone levels	Yao et al., 2022 [151]
PFHpA	Birth	Range Serum (Cord): 0.02–1.17 ng/mL	No association with gross and fine motor, adaptive, language, and social domain scores No association with thyroid hormone levels	Yao et al., 2022 [151]
PFNA	Gestation	Median Plasma (ng/mL): ~0.45–0.49	No association with Cerebral Palsy	Vilhelmsson et al., 2023 [179]
PFTrDA	Gestation (12–16 weeks)	Mean Plasma (ng/mL): 0.1 (PFTrDA)	No association with personal–social skills, gross and fine motor skills, and problem-solving skills	Niu et al., 2019 [153]
PFDA	Gestation (8–16 weeks) and 18 months old	Median Serum (ng/mL): Prenatal: 0.29 (PFDA) Childhood: 0.18 (PFDA)	No association with prenatal and childhood exposure and IQ	Beck et al., 2023 [175]
PFDeA, PFDoDA	Gestation (28–40 weeks)	Median Serum (ng/mL): 0.44 (PFDeA), ~0.37 (PFDoDA)	No association with verbal, performance, and total IQ	Wang et al., 2015 [149]
PFHpS, PFDA, PFOSA	Gestation (6–26 weeks)	Median Plasma: 0.17–2.32 ng/mL (see paper for details)	No association with IQ	Liew et al., 2018 [172]
PFHpS, PFNA, PFDA	Gestation (6–12 weeks)	Range Plasma: ~0.11–0.56 (see paper for details) ng/mL	No association with ADHD or ASD	Liew et al., 2015 [180]
PFHpS, PFNA, PFDA, PFTeDA, PFUnDA	Gestation (22–15 weeks) and at delivery	Median Serum (ug/mL): Prenatal: 0.09–0.6 Infant (Cord): (PFHxS), 0.06–0.45 (see paper for details)	No association with thyroid hormones	Kim et al., 2011 [178]
PFNA, PFDA	Gestation (8–16 weeks) and 18 months	Median Blood (ng/mL): Gestation: 0.29–0.64 18 months: 0.18–0.58 (see paper for details)	No association between prenatal and child exposure and ADHD	Dalsager et al., 2021 [181]
PFNA, PFDA	Gestation (8–16 weeks) and 18 months	Median Serum (ng/mL): Gestation: 0.29–0.65 18 months: 0.18–0.58 (see paper for details)	No association between prenatal and child exposure and language development	Beck et al., 2023 [148]
PFNA	Birth	Median Serum (Cord): ~0.28–0.31 ng/mL	No association with ADHD	Ode et al., 2014 [182]
PFNA	5–18 years	Range Serum: 0.25–24.1 ng/mL	No association with ADHD	Stein and Savitz, 2011 [183]
PFNA	Birth	Range Serum (Cord): 0.08–1.76 ng/mL	No association with gross and fine motor, adaptive, language, and social domain scores No association with thyroid hormone levels	Yao et al., 2022 [151]
PFNA, PFDeA	Gestation (~16 weeks–birth)	Range Serum: 0.1–2.9 (PFNA), 0.1–1.3 (PFDeA) ng/mL	No association with behavioral regulation and executive function	Vuong et al., 2016 [184]
PFNA,	Gestation (18 weeks)	Range Plasma (ng/mL): 0.39–49	No association with ADHD or ASD	Skogheim et al., 2021 [166]
PFNA, PFDA, PFUA, PFDoA, PFBS, PFHpA	Gestation (9–16 weeks)	Median Serum (ng/mL): 0.05–2.16 (see paper for details)	No association with IQ	Wang et al., 2023 [145]
PFNA	Gestation (16–26 weeks)	Median Serum (ug/L):0.9	No association with social responsiveness scores, a measure of ASD	Braun et al., 2014 [185]
PFTrA, PFDoA, PFBA, 8:2Cl-PFESA	Birth	Median Serum (Cord) (ug/L): 0.04–0.25 (see paper for details)	No association with communication scores	Zhou et al., 2023 [152]
PFUA	Birth	Range Serum (Cord): 0.03–0.65 ng/mL	No association with gross and fine motor, adaptive, language, and social domain scores No association with thyroid hormone levels	Yao et al., 2022 [151]

All effects were statistically significant at *p* < 0.05. ↓ refers to a decrease while ↑ refers to an increase in the EDC group(s) compared to a control.

## 6. Phthalate Alternatives and Neurodevelopment

### 6.1. Phthalate Alternatives and Zebrafish

Relatively little research exists looking at the effects of phthalate replacement on neurodevelopment, although what is published suggests that exposure to phthalate replacements can have adverse effects (Table 8). Early ATBC and DINCH exposure led to alterations in locomotion, while ATEC exposure did not [186,187]. Changes in gene expression related to neurodevelopment were seen in zebrafish exposed to ATBC, ATEC, and DINCH [186,187,188]. Effects were also seen in transcriptomics related to thyroid hormone pathways in DGD, DINCH, and GTA, although no effects on thyroid hormone levels were observed [189]. 

### 6.2. Phthalate Alternatives and Rodents

We were only able to identify one study assessing the effects of perinatal phthalate replacement exposure on neurodevelopment and behavior in rodents. Lee et al. observed that exposure to DEHA from GD 15–P21 at ~480–12,000 ppm led to a decrease in hypothalamic granulin precursor protein (grn) mRNA in males and p130 mRNA in females, genes related to brain sexual differentiation. The study also found an effect on sexual behavior, with fewer intromissions and ejaculations by males exposed to the chemical [190].

### 6.3. Phthalate Alternatives and Humans

Human studies examining the effects of phthalate replacements on neurodevelopment are, to our knowledge, limited to DEHTP, DINCH, and DEHT (Table 9). DEHTP exposure during gestation was associated with a decrease in adaptive and cognitive skills in boys, along with a decrease in communication scores in girls [191]. Early DINCH exposure, however, did not have an association with IQ [192]. Gestational exposure to DINCH and DEHT was associated with alterations in thyroid hormones, which could have implications for neurodevelopment [118,193]. 

## 7. Discussion

### 7.1. Bisphenols

Evidence suggests that all BPA analogues pose health risks when it comes to neurodevelopment. BPAF appears to be a particular chemical of concern, with the most adverse effects seen in zebrafish, while BPS causes the fewest disturbances to neurodevelopment. These findings are consistent with evidence suggesting BPAF has high toxicity, teratogenicity, and estrogen receptor binding ability [195,196]. The toxicity level rankings for top bisphenols are BPAF > BPA > BPF > BPS while the estrogenicity rankings are BPAF > BPA = BPF > BPS [195,196]. Rodent and human studies are not as consistent, however. For both, even lower-toxicity bisphenols, such as BPS, caused or were associated with some neurodevelopmental disturbances. BPS, however, did produce null results in many studies. Differences in results between studies may be caused by differences in dosing and timing of EDC exposure in addition to differences in when and how measures (behavioral and molecular) were taken. 

In human studies, authors often excluded bisphenols that were below their defined level of detection. Although this could suggest that these chemicals may be of lesser concern, evidence shows that BPA substitutes are on the rise [39]. Furthermore, the limit of sensitivity of certain assays does not mean that lower exposures are biologically relevant. This demonstrates the need for additional assay development and further monitoring of exposures in humans. It is evident that efforts be put forth to reduce bisphenol exposure overall. 

### 7.2. PFASs

As previously mentioned, the chain length of PFAS is one of the main factors considered regarding toxicity. Comparative studies, however, found that chain length has little to no association with neurodevelopmental endpoints in zebrafish [131,135]. A wide array of PFASs caused neurotoxicity in zebrafish, including GenX, a common short-chain PFAS substitute. While rodent research is lacking, only showing disruption in maternal thyroid levels, human association studies revealed often contradictory findings: PFASs were associated with mixed effects on measures of neurodevelopment and in many cases no effect, possibly due to differing timing of exposure, exposure concentration, and socioenvironmental factors. These findings point to the complexity of endocrine disruptor research and the importance of considering multiple chemicals in epidemiological studies. Furthermore, many endocrine disruptors follow non-monotonic dose responses [197]. Overall, findings demonstrating disruptions in neurodevelopment caused by PFASs at low concentrations, together with limitations in predicting endocrine-disrupting activity based on structural features or length, illustrate the importance of avoiding the regrettable substitution cycle. 

### 7.3. Phthalates

Although research assessing the effects of phthalate substitutes on neurodevelopment is sparse, some studies suggested detrimental effects on neurodevelopment. The zebrafish studies pointed to disruptions in behavior and gene expression in the brain, while human studies found associations with cognition and thyroid hormone levels. A wider body of research is needed to determine whether and how phthalate replacements pose a risk to the developing brain.

### 7.4. Bio-Based Alternatives—Are They Any Safer?

As concerns grow over the environmental and safety repercussions of petroleum-based plastics and plasticizers, bio-based materials have emerged. These include chemicals made from plants, materials intended to degrade naturally, or both. Although they are marketed as safer alternatives, little to no research exists to support or refute this [198]. We were able to find one report that investigated the effects of polylactic acid microplastic (PLA BioMP) exposure in zebrafish larvae. de Oliveria et al. found that 3 and 9 mg/L of PLA BioMPs decreased larvae total locomotion, increased locomotion in the peripheral zone, and increased time spent immobile in the open-field test. They also found a decrease in acetylcholinesterase activity, suggesting a neurotoxic effect of the PLA microplastics [199]. A possible concern is the potential use of soy or other phytoestrogen-based alternatives, which could act on estrogen receptors and alter the developing brain [200]. Thorough testing should be conducted prior to the wide use of bio-based alternatives to ensure minimal effects on neurodevelopment. The use of plants known to act on hormone receptors should also be limited when making bio-based materials.

### 7.5. Conclusions and Recommendations for the Future

**Figure 3:**
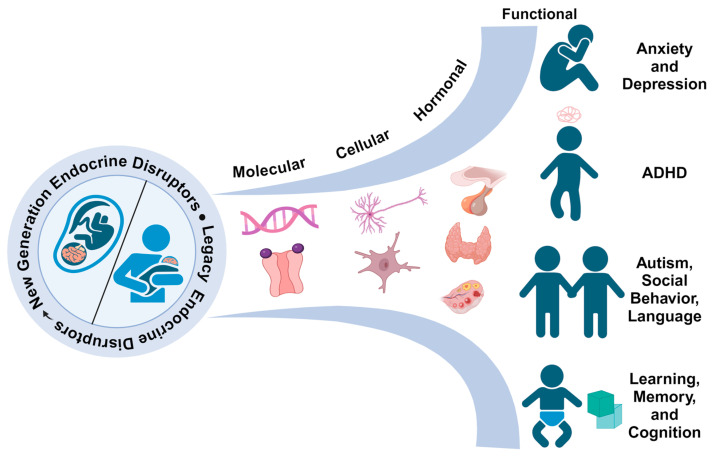
Neurodevelopmental effects of legacy and new-generation endocrine disruptors (created with BioRender.com (accessed on 13 June 2024)).

Legacy and new-generation EDC exposure can have developmental effects on the brain, causing functional changes in humans (Figure 3). As we continue to assess the safety of everyday chemicals and put forward important policy and legislation that restrict the use of EDCs, it is vital that we do not allow companies and corporations to substitute harmful chemicals with equally or more harmful alternatives. Toxicologists and endocrinologists play a key role in formulating guidelines that can help predict and/or identify potential harmful effects of commercial chemicals, and confirmation of the absence of endocrine-mediated and other harmful biological effects should be a prerequisite prior to their circulation in the world. 

## Figures and Tables

**Table 1:** Example substitute bisphenols, PFASs, and phthalate esters.

Bisphenols		
Abbreviation	Common Name	CASRN
BPAF	Bisphenol AF	1478-61-1
BPAP	Bisphenol AP	1571-75-1
BPB	Bisphenol B	77-40-7
BPF	Bisphenol F	620-92-8
BPP	Bisphenol P	2167-51-3
BPS	Bisphenol S	80-09-1
BPS-MAE	-	97042-18-7
BPZ	Bisphenol Z	843-55-0
TCBPF	Tetrachlorobisphenol A	79-95-8
TMBPF	Tetramethylbisphenol F	5384-21-4
**Per- and polyfluoroalkyl substances (PFAS)**		
6:2 Cl-PFESA/F-53B	6:2 chlorinated polyfluorooctane ether sulfonic acid	73606-19-6
8:2 Cl-PFESA/F-53B	8:2 chlorinated polyfluorooctane ether sulfonic acid	83329-89-9
ADONA	4,8-dioxa-3H-perfluorononanoic	919005-14-4
EtFOSAA/NEtFOSAA	2-(N-ethyl-perfluorooctane sulfonamido) acetic acid	2991-50-6
FOSA	Perfluorooctanesulfonamide	754-91-6
GenX/HFPO-DA	Perfluoro-2-methyl-3-oxahexanoic acid	13252-13-6
MeFOSAA/NMeFOSA/ Me-PFOSA-AcOH	2-(N-methyl-perfluorooctane sulfonamido) acetic acid	2355-31-9
PFBA	Perfluorobutanoic acid	375-22-4
PFBS	Perfluorobutanesulfonic acid	375-73-5
PFDA/PFDeA	Perfluorodecanoic acid	335-76-2
PFDoA/PFDoDA	Perfluorododecanoic acid	307-55-1
PFHpA	Perfluoroheptanoic acid	375-85-9
PFHpS	Perfluoroheptanesulfonic acid	375-92-8
PFNA	Perfluorononanoic acid	375-95-1
PFTrDA	Perfluorotridecanoic acid	72629-94-8
PFUA/PFUdA/PFUnA, PFUnDA	Perfluoroundecanoic acid	2058-94-8
**Phthalate esters**		
DINCH	1,2-cyclohexane dicarboxylic acid diisononyl ester	166412-78-8
ATBC	Acetyl tributyl citrate	77-90-7
ATEC	Triethyl 2-acetylcitrate	77-89-4
DGD	Dipropylene glycol dibenzoate	27138-31-4
GTA	Glyceryl triacetate	102-76-1
DEHA/DOA	Di-(2-ethylhexy) adipate	103-23-1
DEHT/DOTP/DEHTP	Di-(2-ethylhexyl) terephthalate	6422-86-2

A list of representative substitute chemicals is provided. Note that additional chemicals not appearing here are discussed in this review.

**Table 4:** Associations between BPA-analogues and neurodevelopment and behavior in humans.

EDC	Time of Exposure Measured	Conc. Measured	Findings	Reference
BPAF	12 months old	<0.01–1.456 ng/mL	↓ Association with social developmental quotient in girls No association with other developmental quotient domains (gross and fine motor, adaptive, and language domains)	Xia et al., 2023 [119]
BPF	6 and 8 years old	Urinary Mean (ug/g creatinine): 6 years: 0.2 8 years: 0.4	↑ Association with ADHD rating scores, higher in girls than boys	Kim et al., 2022 [128]
BPF	Gestation (~10 weeks)	Urinary Mean (ng/mL): 0.16 ng/mL	↓ Association with cognitive function, including verbal comprehension, perceptual reasoning, working memory, and processing speed in boys	Bornehag et al., 2021 [127]
BPF	Gestation (<12 weeks)	Urinary Mean (ng/mL): 0.16	↓ Association with IQ in boys	Tanner et al., 2020 [121]
BPF	Gestation (~13 weeks)	Urinary Mean (ug/L): 0.47	↓ Association with scores of IQ, perceptual reasoning index, and verbal comprehension index among boys	Chen et al., 2023 [120]
BPS	6 and 8 years old	Urinary Mean (ug/g creatinine): 6 years: 0.1 8 years: 0.16	↑ Association with ADHD rating scores, higher in girls than boys	Kim et al., 2022 [128]
BPS	16–19 weeks of pregnancy	Urinary Range (ng/mL): 0.1–3.5	↑ Association with emotionally reactive behaviors in girls	Geiger et al., 2023 [125]
BPS	Gestation (<16 weeks)	Urinary Range (ug/L): ~0.18–0.89	↓ Association with psychomotor development index in boys No association with mental development	Jiang et al., 2020 [123]
TCBPA	Gestation (~13 weeks)	Urinary Range (ug/L): ~0.01–0.08	↑ Association with IQ and perceptual reasoning index scores No association with verbal comprehension	Chen et al., 2023 [120]
BPF	Gestation (<16 weeks)	Urinary Range (ug/L): ~0.38–1.44	No association with infant neurodevelopment (mental development, psychomotor development)	Jiang et al., 2020 [123]
BPF, BPS	Gestation (16–34 weeks)	Urinary Range (ng/mL): 0.1–82.7 (BPF), 0–46.7 (BPS)	No association with nonverbal IQ	van den Dries et al., 2020 [124]
BPS	Gestation (~17 weeks)	Urinary Mean (ug/g creatinine): 0.23	No association with neurodevelopment test assessing cognitive, language, motor, social, emotional, and adaptive skills	Liu et al., 2021 [126]
BPS	Gestation (≤18 weeks)	Urinary Median (ng/mL): 0.34	No association with cord blood or childhood TSH and FT4.	Derakhshan et al., 2021 [118]
BPS	Gestation (~10 weeks)	Urinary Mean (ng/mL): 0.07	No association with cognitive function	Bornehag et al., 2021 [127]
BPS	Gestation (<12 weeks)	Urinary Mean (ng/mL): 0.07	No association with IQ	Tanner et al., 2020 [121]
BPS	Gestation (~13 weeks)	Urinary Range (ug/L creatinine): ~0.03–0.13 (BPAF), ~0–0.002 (BPS)	No association with scores of IQ, perceptual reasoning index, and verbal comprehension index	Chen et al., 2023 [120]
BPS, BPB, BPAP, BPP, BPZ	12 months old	Urinary Range (ng/mL): <0.01–54.605 (BPS), <0.01–0.686 (BPB), <0.01–16.195 (BPAP), <0.01–0.511 (BPP), <0.01–0.512 (BPZ)	No association with developmental quotients (gross and fine motor, adaptive, language, and social domains)	Xia et al., 2023 [119]

All effects were statistically significant at *p* < 0.05. ↓ refers to a decrease while ↑ refers to an increase in the EDC group(s) compared to a control.

**Table 5:** Neurodevelopmental and behavioral effects of non-PFOS, -PFOA, and -PFHxS PFASs in zebrafish.

EDC	Time of Exposure (hpf)	Dose	Findings	Reference
1H,1H,2H,2H-Perfluorohexyl iodide	6–120	0.015–100 μM	↓ Larvae photomotor behavior in the light period No effect on embryonic photomotor behavior	Truong et al., 2022 [135]
1H,1H,8H,8H-Perfluorooctane-1,8-diol	6–120	0.015–100 μM	↓ Embryonic photomotor behavior ↓ Larvae photomotor behavior in the light and dark periods	Truong et al., 2022 [135]
6:2 FTOH	3–120	0.2, 2, 20 μM	↑ Larvae locomotion at 2 μM and 20 μM ↑ Expression of *bdnf* and *ap1s1* at 20 μM (neurodevelopment-related genes)	Annnunziato et al., 2019 [136]
6:2 FTSA	~4–144	0.006–180 μM	↑ Larvae locomotion in dark period at 180 μM No effect on burst activity (movements > 6 mm) or “startle response” (peak distance − mean distance)	Menger et al., 2020 [134]
Six PFASs, including GenX, PFHpS (see paper for details)	8–120	0.35–0.92 μM	↓ Larvae photomotor behavior in light period No effect on embryonic photomotor behavior and larvae startle response	Rericha et al., 2021 [131]
8:8 PFPiA	4–144	0.0116–5.79 μM	↓ Larvae locomotion at 0.343–5.79 μM in the dark periods ↑ Expression of *chrb*, *dio3a*, and *tshr* (HPT axis) at 5.79 μM; No effect on expression of *dio3b*, *thraa*, and *thrb* (axis) ↓ Expression of *elavl3* at 1.35–5.79 μM (neurodevelopment) No effect on expression of *mbp*, *syn2a*, *shha*, and *tuba1* (neurodevelopment)	Kim et al., 2020 [137]
DiSAMPAP	8–120	0.20 μM	↑ Larvae photomotor behavior in dark period No effect on embryonic photomotor behavior and larvae startle response	Rericha et al., 2021 [131]
Five PFASs (see paper for details)	6–120	0.015–100 μM	↑ Embryonic photomotor behavior No effect on larvae photomotor behavior	Truong et al., 2022 [135]
Five PFASs (see paper for details)	6–120	0.015–100 μM	↓ Larvae photomotor behavior in the dark period No effects on embryonic photomotor behavior	Truong et al., 2022 [135]
FOSA	6–120	0.015–100 μM	↑ Embryonic photomotor behavior ↓ Larvae photomotor behavior in the dark period	Truong et al., 2022 [135]
Four PFASs (see paper for details)	6–120	0.015–100 μM	↓ Embryonic photomotor behavior No effect on larvae photomotor response	Truong et al., 2022 [135]
Four PFASs, including PFUA (see paper for details)	6–120	0.015–100 μM	↑ Larvae photomotor behavior in the light and dark periods No effect on embryonic photomotor behavior	Truong et al., 2022 [135]
GenX	6–168	0.1–10,000 μg/L	↑ Expression of *elavl3*, *gap43*, *tubb* (neuron differentiation and growth), *gfap* (astrocytes); ↓ expression of *manf* (neurotrophic signals) at 0.1 and 1 μg/L (other doses not measured) No effect on expression of *mbp* (axon function), *nestin* (neuron structure) at 0.1 and 1 μg/L (other doses not measured) ↓ Larvae locomotion in dark period at 1000 μg/L; in light periods at 100 μg/L No effect in light-dark preference test (anxiety)	Ivantsova et al., 2023 [129]
GenX	1–120	4–4000 ppb	↑ Larvae locomotion at 40–4000 ppb in the dark periods; ↓ at 4000 ppb in the light period ↑ Dopamine at 40 ppb	Wasel et al., 2023 [130]
Methyl 3H-perfluoroisopropyl ether	6–120	0.015–100 μM	↓ Larvae photomotor behavior in the light and dark period	Truong et al., 2022 [135]
PFBA	8–120	0.98–98 μM	↑ Larvae photomotor behavior in the light period ↑ Larvae photomotor behavior at all doses in the dark period and at 2.49, 6.32, and 34.3–98 μM in the light period No effect on embryonic photomotor behavior and larvae startle response	Rericha et al., 2021 [131]
PFBA	1–120	23,360–46,720 μM	↓ Larvae locomotion at 0.4 ppb in the light; at 400, 4000 ppb in the dark; ↑ at 4 in the dark. ↑ Head width at 400 ppb ↑ Enrichment of pathways associated with neurological disorders, behavior, and nervous system development and function	Wasel et al., 2022 [132]
PFBS	1–120	4–4000 ppb	↑ Larvae locomotion at 4–4000 ppb in the dark period; at 40–4000 ppb in the light period ↓ Dopamine at 400 ppb	Wasel et al., 2023 [130]
PFBS	~4–144	10–3000 mg/L	↓ Larvae locomotion at 1000 μg/L (3000 mg/L not measured)	Ulhaq et al., 2013 [133]
PFDA	8–120	0–100 μM	No effect on embryonic or larvae photomotor behavior and larvae startle response ↑ Larvae photomotor behavior in the dark period at 2.49 and 34.3 μM, and in the light at 2.5 μM ↓ at 16.07 and 100 μM	Rericha et al., 2021 [131]
PFDMMOBA	4–168	100–300 ppm	↑ Listing (falling to one side)	Gebreab et al., 2020 [138]
PFDoA	~4–120	0, 0.24, 1.2, 6 mg/L	↓ Larvae locomotion at all doses ↓ GFP neurons at 6 mg/L ↓ ACh at 6 mg/L and AChE activity at 1.2 or 6 mg/L ↑ Dopamine at 1.2 and 6 mg/L ↓ Expression *α1-tubulin*, *gap43, gfap*, *shha*, *syn2a*, *ache* at 6 mg/L; *mbp*, *elavl3* at 1.2 and 6 mg/L; ↑ *manf* at 6 mg/L	Guo et al., 2018 [139]
PFECHS, PFPrS	8–120	0.35–0.50 μM	↓ Larvae startle response No effect on embryonic or larvae photomotor behavior	Rericha et al., 2021 [131]
PFHpA	~4–144	0.002–89 μM	↑larvae locomotion in light period at 89 μM No effect on burst activity (movements > 6 mm) or “startle response” (peak distance − mean distance)	Menger et al., 2020 [134]
PFHpA	8–120	0.69 μM	No effect on embryonic photomotor behavior and larvae startle response ↑ Larvae photomotor behavior at 2.51 and 6.39 μM ↓ at 100 μM in the dark period	Rericha et al., 2021 [131]
PFHpS	0–120	1.7–31.4 μM	↑ Larvae locomotion at 3.1 and 5.5 μM in the light and dark periods	Gaballah et al., 2020 [140]
PFHpS, PFOS-K, PFTrDA	6–120	0.015–100 μM	↑ Larvae photomotor behavior in the light period; ↓ in the dark No effect on embryonic photomotor behavior	Truong et al., 2022 [135]
PFHxA	8–120	0.80 μM	↑ Area under curve analysis (change in movement from light to dark periods) in larvae photomotor behavior ↓ Larvae photomotor behavior at 2.46, 6.32, 34.3, and 73.3 μM in the dark period and ↑ at 2.46 and 16.07 μM in the light period No effect on embryonic photomotor behavior and larvae startle response	Rericha et al., 2021 [131]
PFHxA	0–120	4.4–80.0 μM	↑ Larvae locomotion at 25.1 μM in the light period and at 14 and 25.1 μM in the dark period	Gaballah et al., 2020 [140]
PFHxA	3–120	0.2, 2, 20 μM	No effect on larvae locomotion ↑ Expression of *bdnf* at 20 μM; *ap1s1* at 2 and 20 μM (neurodevelopment-related genes)	Annnunziato et al., 2019 [136]
PFHxA	1–120	15,921.03–31,842.06 μM	No effect on larvae locomotion ↓ Head length at 40 and 400 ppb No found enrichment of pathways associated with the nervous system and behaviors	Wasel et al., 2022 [132]
PFNA	~4–144	0.03–10 mg/L	↓ Larvae locomotion at 10 mg/L	Ulhaq et al., 2013 [133]
PFNA	3–120	1–2.0 μM	↓ Larvae locomotion at all doses ↑ Time spent in well center at 0.2 and 2.0 μM	Jantzen et al., 2016 [141]
PFNA	~4–144	0.001–200 μM	↓ Larvae locomotion in light period at 48uM (higher doses not measured) ↑ Burst activity (movements > 6 mm) in the dark period at 15 and 48 μM	Menger et al., 2020 [134]
PFNA	8–120	0–100 μM	↓ Larvae startle response at 0.54 μM ↓ Larvae photomotor behavior at 33.95 μM in the dark period and at 2.46 and 33.95 μM in the light period ↑ in the light period at 0.97 and 72.56 μM No effect on embryonic photomotor behavior	Rericha et al., 2021 [131]
PFO2DA	4–168	5–100 ppm	↑ Listing (falling to one side)	Gebreab et al., 2020 [138]
PFO3DA	4–168	25–200 ppm	↑ Listing (falling to one side)	Gebreab et al., 2020 [138]
PFO3TDA	4–168	1–40 ppm	↑ Listing (falling to one side)	Gebreab et al., 2020 [138]
PFPeA	8–120	0.97–97 μM	↑ Larvae photomotor behavior at 15.91, 33.95, and 97 μM in the dark period and 6.26–33.95 μM in the light period ↓Larvae photomotor behavior in the light period at 0.95 μM No effect on embryonic photomotor behavior and larvae startle response	Rericha et al., 2021 [131]
PFPeS	0–120	3.1–56.0 μM	↑ Larvae locomotion at 3.1 and 5.5 μM in the light periods	Gaballah et al., 2020 [140]
PFTrDA	8–120	0–35 μM	↓ Larvae photomotor behavior at 10 μM in the dark period, 35 μM at light period, and ↑ at 10 μM in the light period. No effect on embryonic photomotor behavior and larvae startle response	Rericha et al., 2021 [131]
PFUA	8–120	0–75.0 μM	No effect on embryonic photomotor behavior and larvae startle response ↓ Larvae photomotor behavior in dark period at 52.8 μM and in light period at 0.96 and 2.44 μM	Rericha et al., 2021 [131]
Six PFASs, including PFBS and PFDoA (see paper for details)	8–120	0.25–0.75 μM (see paper for details)	↑ Larvae photomotor behavior in light period No effect on embryonic photomotor behavior and larvae startle response	Rericha et al., 2021 [131]
TFAA	~4–144	10–3000 mg/L	↓ Larvae locomotion at 1000 and 3000 mg/L	Ulhaq et al., 2013 [133]
Three PFASs (see paper for details)	6–120	0.015–100 μM	↑ Larvae photomotor behavior in the light period No effect on embryonic photomotor behavior	Truong et al., 2022 [135]
Two PFASs (see paper for details)	6–120	0.015–100 μM	↑ Larvae photomotor behavior in the dark No effect on embryonic photomotor behavior	Truong et al., 2022 [135]
Two PFASs (see paper for details)	6–120	0.015–100 μM	↓ Larvae photomotor behavior in the dark period; ↑ in the light period No effect on embryonic photomotor behavior	Truong et al., 2022 [135]
107 PFASs, including PFBA, PFBS, PFNA, GenX, PFDA, PFHpA, (see paper for details)	6–120	0.015–100 μM	No effect on embryonic or larvae photomotor behavior	Truong et al., 2022 [135]
33 PFASs, including EtFOSAA, PFNA, (see paper for details)	8–120	0.27–1.03 μM (see paper for details)	No effect on embryonic or larvae photomotor behavior and larvae startle response	Rericha et al., 2021 [131]
ADONA, GenX	0–120	4.4–80.0 μM	No effect on larvae locomotion	Gaballah et al., 2020 [140]
GenX	5– 120	0.1–150 μM	No effect on startle response to light	Satbhai et al., 2022 [142]
PFBA, PFDA	~4–144	10–3000 mg/L	No effect on larvae locomotion	Ulhaq et al., 2013 [133]
PFBS	0–5dpf	5.5–100.0 μM	No effect on larvae locomotion	Gaballah et al., 2020 [140]
PFESA1	0–120	4.4–80.0 μM	No effect on larvae locomotion	Gaballah et al., 2020 [140]
PFHxA, PFPeA, PFBS	~4–144	0.002–84 μM (see paper for details)	No effect on larvae locomotion, burst activity (movements > 6 mm), or “startle response” (peak distance − mean distance)	Menger et al., 2020 [134]
PFMOBA PFO2HPA GenX	4–168	25–200 ppm	No effect on listing (falling to one side)	Gebreab et al., 2020 [138]

All effects were statistically significant at *p* < 0.05. ↓ refers to a decrease while ↑ refers to an increase in the EDC group(s) compared to a control.

**Table 6:** Neurodevelopmental and behavioral effects of non-PFOS, -PFOA, and -PFHxS PFASs in rodents.

EDC	Animal	Time of Exposure	Dose	Findings	Reference
GenX	Rats	GD14–18	62.5–500 mg/kg or 1–30 mg/kg	↓ maternal T3 at 30–500 mg/kg; T4 at 125–500 mg/kg	Conley et al., 2019 [143]
GenX	Rats	GD1.5–11.5 or GD 1.5–17.5	2, 10 mg/kg	↑ placental T4 at 10 mg/kg	Blake et al., 2020 [144]

All effects were statistically significant at *p* < 0.05. ↓ refers to a decrease while ↑ refers to an increase in the EDC group(s) compared to a control.

**Table 8:** Neurodevelopmental and behavioral effects of phthalate alternatives in zebrafish.

EDC	Time of Exposure (hpf)	Dose	Findings	Reference
ATBC	0–96	0.03–300 μg/L	↓ Locomotion at 300 μg/L in the light and dark phase ↓ AchE at 300 μg/L No effect on dopamine ↓ Expression of *ache*, *gap43*, *mbpa*, and *syn2a* at 300 μg/L (related to neurodevelopment) No effect on expression of *gfap*, *shha*, *tuba1b* (genes related to neurodevelopment)	Yun et al., 2024 [186]
ATBC	4–120	10–1000 μg/L	↓ Expression of *tshβ* at 19 and 194.5 μg/L; *trα* at 194.5 μg/L; *dio1* at 194.5 μg/L; *dio2* at 71 and 194.5 μg/L (genes related to neurodevelopment)	Horie et al., 2022 [188]
ATEC	0–96	0.03–300 μg/L	No effect on locomotion No effect on AChE or dopamine ↓ Expression of *ache* and *mbpa* at 300 μg/L (genes related to neurodevelopment) No effect on expression of *gap43*, *gfap*, *shha*, *syn2a*, *tuba1b* (genes related to neurodevelopment)	Yun et al., 2024 [186]
DGD	8–48	0.000001–1 μM	No effect on thyroid hormone levels ↑ Association with transcriptomics adverse outcome pathway related to thyroid hormone	Tan et al., 2023 [189]
DINCH	2–144	0.01–10 μM	↑ Locomotion at 0.1–10 μM during the light period ↑ Expression of *pcsk9* at all doses, *dmrt3a* at 0.1–10 μM, *hmgcs1* at 10 μM, *mbpa* at 1, 10 μM; ↓ *dhcr7* at all doses (genes related to cholesterol biosynthesis, myelin, neurodevelopment)	Saad et al., 2021 [187]
DINCH	8–48	10–1000 μg/L	No effect on thyroid hormone levels ↑ Association with transcriptomics adverse outcome pathway related to thyroid hormone	Tan et al., 2023 [189]
GTA	8–48	0.001–1000 μM	No effect on thyroid hormone levels ↑ Association with transcriptomics adverse outcome pathway related to thyroid hormone	Tan et al., 2023 [189]

All effects were statistically significant at *p* < 0.05. ↓ refers to a decrease while ↑ refers to an increase in the EDC group(s) compared to a control.

**Table 9:** Associations with phthalate alternatives and neurodevelopment and behavior in humans.

EDC	Time EDC Measured	Concentration Measured	Findings	Reference
DEHTP	Gestation (~14 weeks)	Mean Urine (metabolites, ng/mL): MECPTP: 28.76 MEHHP: 5.72	↓ association with adaptive and cognitive domain in boys; association with communication scores in girls	Park et al., 2023 [191]
DINCH	7–11 years old	Mean Urine (Biomarkers, μg/L): OH-MINCH: 2.3–3.6 cx-MINCH: 1.1–2.3	No association with IQ	Rosolen et al., 2022 [192]
DINCH	Gestation (>14 weeks)]	Mean Urine (biomarkers, ng/mL): MOiNCH: 0.25	↑ association with TT3; ↓ association with TT4/TT3 ratio	Derakhshan et al., 2021 [194]
DINCH	Gestation (~14 weeks)	Mean Urine (metabolites, ng/mL): MHinCH: 0.47 MCOCH: 0.47	No association with thyroid hormones (TSH, T3, T4, T3/T4 ratio)	Cathey et al., 2019 [193]
DEHT	Gestation (~14 weeks)	Mean Urine (metabolites, ng/mL): MECPTP: 20.5 MEHHTP: 3.72	↑ association with T3, T3/T4 ratio No association with TSH or T4	Cathey et al., 2019 [193]

All effects were statistically significant at *p* < 0.05. ↓ refers to a decrease while ↑ refers to an increase in the EDC group(s) compared to a control.

## Data Availability

Not applicable.
